# Whole-genome comparison using complete genomes from *Campylobacter fetus* strains revealed single nucleotide polymorphisms on non-genomic islands for subspecies differentiation

**DOI:** 10.3389/fmicb.2024.1452564

**Published:** 2024-09-12

**Authors:** Chian Teng Ong, Patrick. J. Blackall, Gry B. Boe-Hansen, Sharon deWet, Ben J. Hayes, Lea Indjein, Victoria Korolik, Catherine Minchin, Loan To Nguyen, Yusralimuna Nordin, Hannah Siddle, Conny Turni, Bronwyn Venus, Mark E. Westman, Zhetao Zhang, Ala E. Tabor

**Affiliations:** ^1^Queensland Alliance for Agriculture and Food Innovation, Centre for Animal Science, The University of Queensland, St Lucia, QLD, Australia; ^2^Queensland Alliance for Agriculture and Food Innovation, Centre for Animal Science, The University of Queensland, Dutton Park, QLD, Australia; ^3^School of Veterinary Science, The University of Queensland, Gatton, QLD, Australia; ^4^Department of Agriculture and Fisheries, Biosecurity Sciences Laboratory, Coopers Plains, QLD, Australia; ^5^Institute for Glycomics, Griffith University, Nathan, QLD, Australia; ^6^Department of Agriculture and Fisheries, Agri-Science Queensland, Animal Science, Dutton Park, QLD, Australia; ^7^Department of Primary Industries, Elizabeth Macarthur Agricultural Institute, Menangle, NSW, Australia; ^8^School of Chemistry and Molecular Biosciences, The University of Queensland, St Lucia, QLD, Australia

**Keywords:** *Campylobacter fetus*, complete genomes, subspecies, SNPs, genome comparison, glycine, veterinary science

## Abstract

**Introduction:**

Bovine Genital Campylobacteriosis (BGC), caused by *Campylobacter fetus* subsp. venerealis, is a sexually transmitted bacterium that significantly impacts cattle reproductive performance. However, current detection methods lack consistency and reliability due to the close genetic similarity between *C. fetus* subsp. venerealis and *C. fetus* subsp. fetus. Therefore, this study aimed to utilize complete genome analysis to distinguish genetic features between *C. fetus* subsp. venerealis and other subspecies, thereby enhancing BGC detection for routine screening and epidemiological studies.

**Methods and results:**

This study reported the complete genomes of four *C. fetus* subsp. fetus and five *C. fetus* subsp. venerealis, sequenced using long-read sequencing technologies. Comparative whole-genome analyses (*n* = 25) were conducted, incorporating an additional 16 complete *C. fetus* genomes from the NCBI database, to investigate the genomic differences between these two closely related *C. fetus* subspecies. Pan-genomic analyses revealed a core genome consisting of 1,561 genes and an accessory pangenome of 1,064 genes between the two *C. fetus* subspecies. However, no unique predicted genes were identified in either subspecies. Nonetheless, whole-genome single nucleotide polymorphisms (SNPs) analysis identified 289 SNPs unique to one or the *C. fetus* subspecies. After the removal of SNPs located on putative genomic islands, recombination sites, and those causing synonymous amino acid changes, the remaining 184 SNPs were functionally annotated. Candidate SNPs that were annotated with the KEGG “Peptidoglycan Biosynthesis” pathway were recruited for further analysis due to their potential association with the glycine intolerance characteristic of *C. fetus* subsp. venerealis and its biovar variant. Verification with 58 annotated *C. fetus* genomes, both complete and incomplete, from RefSeq, successfully classified these seven SNPs into two groups, aligning with their phenotypic identification as CFF (*Campylobacter fetus* subsp. fetus) or CFV/CFVi (*Campylobacter fetus* subsp. venerealis and its biovar variant). Furthermore, we demonstrated the application of mraY SNPs for detecting *C. fetus* subspecies using a quantitative PCR assay.

**Discussion:**

Our results highlighted the high genetic stability of *C. fetus* subspecies. Nevertheless, *Campylobacter fetus* subsp. venerealis and its biovar variants encoded common SNPs in genes related to glycine intolerance, which differentiates them from *C. fetus* subsp. fetus. This discovery highlights the potential of employing a multiple-SNP assay for the precise differentiation of *C. fetus* subspecies.

## Introduction

*Campylobacter* spp. are Gram-negative, microaerophilic bacteria that are generally curved-shaped rods (Sebald and Veron, 1963; Smibert, 1981). *Campylobacter fetus* was first described as *Vibrio fetus* in 1919 (Smith and Taylor, 1919), and it was identified as a pathogenic species that can cause disease in humans and a number of other hosts (Smibert, 1981). There are three subspecies of *C. fetus*—*C. fetus* subsp. *fetus, C. fetus* subsp. *testudinum*, and *C. fetus* subsp. *venerealis* (Tu et al., 2004; Smibert, 1978). These *C. fetus* subspecies form a distinct host dichotomy (Gilbert et al., 2016; Tu et al., 2001). Specifically, the host for *C. fetus* subsp. *testudinum* is primarily reptiles (Fitzgerald et al., 2014), while *C. fetus* subsp. *fetus* and *C. fetus* subsp. *venerealis* are primarily associated with mammals (Marsh and Firehammer, 1953).

*Campylobacter fetus* subsp. *fetus* has been isolated from a broader range of hosts than *C. fetus* subsp. *venerealis*, including cattle, sheep, and humans, mainly from the gastrointestinal tract and occasionally from aborted fetuses (Smibert, 1978). The epidemiology of *C. fetus* subsp. *fetus* infection features persistent but mild infection and sporadic abortion only. In contrast, the colonization of *C. fetus* subsp. *venerealis* is highly host- and niche-specific, as this organism is confined to the bovine genital tract (Penner, 1988; Hoffer, 1981; Hum, 1996; OIE, 2019). *Campylobacter fetus* subsp. *venerealis* is recognized as the etiologic agent of Bovine Genital Campylobacteriosis (BGC), which is a venereal disease associated with low herd fertility and high economic loss across multiple geographical locations (Sprenger et al., 2012). Transmission of *C. fetus* subsp. *venerealis* is through natural mating with asymptomatic bulls or insemination with contaminated semen or equipment. The persistent infection of this subspecies in the female reproductive tract results in BGC, which is often manifested as infertility, embryonic loss, and abortions in the first part of pregnancy (Mshelia et al., 2010).

The expression of Surface Array Proteins (SAP) correlates with serovars of the *C. fetus* subsp. *fetus* (serovars A, B, and AB), *C. fetus* subsp. *venerealis* (serovar A), and *C. fetus* subsp. *testudinum* (serovars A, B and AB). However, it is not distinctive at the subspecies level (Moran et al., 1994; Perez-Perez et al., 1986; Dworkin et al., 1995). Nonetheless, *C. fetus* subsp. *testudinum* has been demonstrated to be genetically distinct from its two closely related subspecies (Fitzgerald et al., 2014; Dingle et al., 2010). A phylogenetic reconstruction, which involved 61 *C. fetus* genomes, revealed a barrier to lateral gene transfer between *C. fetus* subsp. *testudinum* and the other *C. fetus* subspecies (Gilbert et al., 2016). Additionally, several genetic features segregating the reptile-associated *C. fetus* subsp. *testudinum* from mammal-associated *C. fetus* were reported, including the exclusive presence of a putative locus encoding for tricarballylate catabolism pathway in *C. fetus* subsp. *testudinum* (Gilbert et al., 2016). Biomarkers based on proteotyping have also been identified for the differentiation of *C. fetus* subsp. *testudinum* from the other two *C. fetus* subspecies (Emele et al., 2019).

In comparison, despite their differences in niche specificity, biochemical properties, and pathogenicity, there is no universally recognized method for the differentiation between *C. fetus* subsp. *fetus* and *C. fetus* subsp. *venerealis* (Nadin-Davis et al., 2021; van der Graaf-van Bloois et al., 2014). The two biochemical tests described in the OIE Terrestrial Manual (OIE, 2019) for differentiating the *C. fetus* subspecies are the 1% glycine tolerance test and H_2_S production from cysteine-rich medium, with *C. fetus* subsp. The *fetus* was positive for both tests, while *C. fetus* subsp. *venerealis* is negative in both tests. Genome analysis has also confirmed the partial deletion of a putative cysteine transporter in *C. fetus* subsp. *venerealis* strains (van der Graaf-van Bloois et al., 2016a). However, the reliability of the biochemical tests for subspecies differentiation is complicated by the presence of the biovar *C. fetus* subsp. *venerealis* bv. intermedius, which possesses phenotypic characteristics from both *C. fetus* subsp. *fetus* and *C. fetus* subsp. *venerealis* (Sprenger et al., 2012; Iraola et al., 2013). *Campylobacter fetus* subsp. *venerealis* bv. intermedius has been isolated from both bovine intestinal and genital tracts and is glycine intolerant, which is typical of *C. fetus* subsp. *venerealis* but H_2_S positive as is typical of *C. fetus* subsp. *fetus* (OIE, 2019).

Amplified fragment length polymorphism (AFLP) and pulsed-field gel electrophoresis (PFGE) were shown to be effective in correlating the phenotypic and genotypic characteristics of *C. fetus* subsp. *fetus* and *C. fetus* subsp. *venerealis*; the high labor costs and the difficulty in isolating pure cultures of *C. fetus* from clinical samples required for these methods render them not ideal for routine testing by diagnostic laboratories (On and Harrington, 2001; van Bergen et al., 2005a; Wagenaar et al., 2001). Other discriminatory methods, for example, serotyping (Moran et al., 1994; Perez-Perez et al., 1986), DNA hybridization tests (Harvey and Greenwood, 1983; Basden et al., 1968), and protein banding patterns (Vandamme et al., 1990) were not able to accurately distinguish *C. fetus* subsp. *fetus* from *C. fetus* subsp. *venerealis*. Genomic studies suggested that these two *C. fetus* subspecies share a high level of genome synteny, with *C. fetus* subsp. *venerealis* possessing increased genome length and plasticity compared to *C. fetus* subsp. *fetus* (van der Graaf-van Bloois et al., 2014; Ali et al., 2012; Kienesberger et al., 2014). The *C. fetus* subsp. *venerealis* adaptation was attributed to the presence of hypervariable regions, pathogenicity islands and the acquisition of transposable elements, including prophages, transposons, and plasmids encoding for virulence factors (Nordin, 2013). Subspecies discrimination between *C. fetus* subsp. *fetus* and *C. fetus* subsp. *venerealis* using molecular methods has been attempted. For example, a multiple locus sequence typing (MLST) scheme based on the seven housekeeping genes categorized sequence type 4 (ST-4) exclusively with *C. fetus* subsp. *venerealis* (van Bergen et al., 2005b). However, ST-4 was later found to also be present in *C. fetus* subsp. *fetus* strain H1-UY (Iraola et al., 2015).

Several polymerase chain reaction (PCR) targets, including the plasmid partition protein A (*par*A; Hum et al., 1997) and the putative VirB6 protein truncated by the insertion element (*ISCfe1*; Abril et al., 2007), have been developed for the subspecies identification after investigating the different pathogenicity of *C. fetus* subspecies. The early research proposed that *C. fetus* subsp. *venerealis* displays a higher level of pathogenicity because of the genomic island in its genome (Iraola et al., 2012; Moolhuijzen et al., 2009). Several molecular targets were developed to target the genomic island, which encodes a type IV secretion system (T4SS). However, these assays with transfer-associated genes lacked specificity later when they were tested against multiple strains from both subspecies and related *Campylobacter* strains (Hum et al., 1997; Abril et al., 2007). For instance, the *par*A gene was not detected in 10 *C. fetus* subsp. *venerealis* in the previous study (Silva et al., 2021). Moreover, the previous PCR tests using various molecular targets on T4SS, including VirB6, also did not consistently align the *C. fetus* strains with their phenotypic identification (Nadin-Davis et al., 2021; Abril et al., 2007; van der Graaf-van Bloois et al., 2016b). There was no strong evidence of subspecies misidentification with *ISCfe1*, except its absence in *C. fetus* subsp. *venerealis* CCUG 34111 (Abril et al., 2007). Since T4SS-encoding regions are not exclusive to *C. fetus* subsp. *venerealis* and were found present in *C. fetus* subsp. *fetus* (Kienesberger et al., 2014; van der Graaf-van Bloois et al., 2016b) as well as other related *Campylobacter* bacteria, including *C. jejuni, C. lari*, and *C. coli* (Moolhuijzen et al., 2009), the reliability of *ISCfe1*, which is the insertion element truncated the T4SS VirB6 protein, for *C. fetus* subsp. *venerealis*, detection should be tested with a large number of *Campylobacter* strains from multiple continents. Another successful diagnostic test was developed using the L-Cys transporter-deletion polymorphism as the potential marker for H_2_S-positive *C. fetus* strains (van der Graaf-van Bloois et al., 2016a; Farace et al., 2019). However, this PCR assay was not able to capture the intermedius biovar variant, which is positive for H_2_S production (Farace et al., 2019, 2021).

The results from the previous study have suggested that the full genome sequence of the *C. fetus* subsp. *venerealis* and its biovars from different geographical continents will benefit the *C. fetus* subsp. *venerealis* detection research (Moolhuijzen et al., 2009). The genome completeness was demonstrated to be beneficial for whole-genome comparisons, particularly genomic regions with low coverage, high GC content, and/or high repetitiveness (Malmberg et al., 2019; Goldstein et al., 2019). Using complete genomes can therefore prevent the identification of false positives that arise from analyzing incomplete genomes, as missing data can lead to incorrect conclusions (Ribeiro et al., 2015; Ceres et al., 2022).

Therefore, in this study, we aimed to generate closed and complete genomes for nine *C. fetus* strains, including four *C. fetus* subsp. *fetus*, three *C. fetus* subsp. *venerealis*, and two *C. fetus* subsp. *venerealis* bv. intermedius, using Oxford Nanopore Technologies (ONT) long-read sequencing. Using these closed genomes in addition to the complete *C. fetus* genomes available on NCBI RefSeq, we conducted a whole-genome comparison to investigate the phylogenetic relationship between *C. fetus* subsp. *fetus* and *C. fetus* subsp. *venerealis* by examining their genome identity, differentially expressed gene orthologs, and single nucleotide polymorphisms (SNPs).

## Materials and methods

### *Campylobacter fetus* strains and genomes

The bacterial strains sequenced in this study were from three different culture collections, the details of which are summarized in Table 1. Briefly, M20-08756/1A and M20-04752/1B were kindly gifted by the Department of Primary Industries in New South Wales, Australia, while isolates BT268/06 and BT376/03 were kindly gifted by the Institute for Glycomics at Griffith University in Queensland, Australia. The other isolates, including A8, 957, 76223, 924, and 926, were in-house isolates at the Queensland Alliance for Agriculture and Food Innovation at the University of Queensland, Australia, which were previously isolated from a local abattoir (Indjein, 2013). In total, four *C. fetus* subsp. *fetus* (M20-08756/1A, M20-04752/1B, BT268/06, and BT376/03), three *C. fetus* subsp. *venerealis* (A8, 957, and 76223), and two *C. fetus* subsp. *venerealis* bv. intermedius (924 and 926) strains were used in this study (Table 1). These strains were previously phenotyped using the standard OIE biochemical assays, and their subspecies identity was confirmed by cpn60 gene sequencing (Nordin, 2013; Indjein, 2013; Koya, 2016). The type strains for *C. fetus* subsp. *fetus* (ATCC 27374^T^) and *C. fetus* subsp. *venerealis* (ATCC 19438^T^), which had their complete genome published on the National Center for Biotechnology Information (NCBI) database, were also sequenced in this study to serve as internal controls. The cultures were stored at −80°C and were revived by culturing on the tryptone soya agar supplemented with 5% defibrinated sheep blood (Thermo Scientific, Delaware, USA) under micro-aerophilic conditions at 37°C for 48 h. Colonies of each bacterial strain were resuspended in sterile phosphate-buffered saline to reach an optical density measured at a wavelength of 600 nm (OD_600_) to yield ~1 x 10^9^ colony-forming units per mL (cfu/mL). Genomic DNA extraction of the pure bacterial culture was conducted using the Genomic-tip extraction kit (QIAGEN, Hilden, Germany). The quantity and quality of extracted gDNA were assessed using a Qubit^TM^ 4 fluorometer (Thermo Scientific, Delaware, USA) and pulsed-field gel electrophoresis (Pippin Pulse, Sage Science, Massachusetts, USA).

**Table 1:** List of *Campylobacter fetus* strains sequenced and analyzed in this study.

**(A) Strains analyzed in this study**						
**Strain**	**Accession**	**Organism**	**Country of origin**	**Year of isolation**	**Isolation source**	**Collection**
M20-08756/1A	GCF_032594895.1	*Campylobacter fetus* subsp. *fetus*	New Zealand	1986	Ovine (fetal stomach contents)	1
BT376/03	GCA_030544625.1	*Campylobacter fetus* subsp. *fetus*	United Kingdom	2003	Bovine	2
BT268/06	GCA_030544645.1	*Campylobacter fetus* subsp. *fetus*	United Kingdom	2006	Ovine	2
M20-04752/1B	GCF_032594815.1	*Campylobacter fetus* subsp. *fetus*	Australia	2020	Ovine (fetal liver)	1
A8	CP075536-CP075537	*Campylobacter fetus* subsp. *venerealis*	Australia	2011	Bovine	3
957	GCF_030544565.1	*Campylobacter fetus* subsp. *venerealis*	Australia	2011	Bovine (bull prepuce)	3
76223	GCF_030544545.1	*Campylobacter fetus* subsp. *venerealis*	Australia	2012	Bovine (aborted fetus)	3
924	GCF_030544605.1	*Campylobacter fetus* subsp. *venerealis* bv. intermedius	Australia	2011	Bovine (bull prepuce)	3
926	GCF_030544585.1	*Campylobacter fetus* subsp. *venerealis* bv. intermedius	Australia	2011	Bovine (bull prepuce)	3
**(B) Strains sequenced in previous studies**						
**Strain**	**Accession**	**Organism**	**Country of origin**	**Year of isolation**	**Isolation source**	
ATCC 27374^T^	GCA_900475935.1	*Campylobacter fetus* subsp. *fetus*	France	1952	Ovine (fetus)	
82-40	GCA_000015085.1	*Campylobacter fetus* subsp. *fetus*	USA	1982	Human	
00A031	GCA_011600945.2	*Campylobacter fetus* subsp. *fetus*	Canada	2000	Bovine (bull prepuce)	
02A725-35A	GCA_011600855.2	*Campylobacter fetus* subsp. *fetus*	Canada	2002	Bovine (bull prepuce)	
04-554	GCA_000759485.1	*Campylobacter fetus* subsp. *fetus*	Argentina	2004	Bovine (aborted fetus)	
09A980	GCA_011600995.2	*Campylobacter fetus* subsp. *fetus*	Canada	2009	Bovine (bull prepuce)	
INIA/17144	GCA_007723545.1	*Campylobacter fetus* subsp. *fetus*	Uruguay	2017	Ovine (placenta)	
ATCC 19438^T^	GCA_008271385.1	*Campylobacter fetus* subsp. *venerealis*	United Kingdom	1962	Bovine (vaginal mucus)	
84-112	GCA_000967135.1	*Campylobacter fetus* subsp. *venerealis*	USA	1984	Bovine	
97-608	GCA_000759515.1	*Campylobacter fetus* subsp. *venerealis*	Argentina	1987	Bovine	
08A948-2A	GCA_011601005.2	*Campylobacter fetus* subsp. *venerealis*	Canada	2008	Bovine (bull prepuce)	
08A1102-42A	GCA_011600845.2	*Campylobacter fetus* subsp. *venerealis*	Canada	2008	Bovine (bull prepuce)	
ADRI545	GCA_011601375.2	*Campylobacter fetus* subsp. *venerealis* bv. intermedius	Australia	1984	Bovine (reproductive tract)	
ADRI1362	GCA_011600955.2	*Campylobacter fetus* subsp. *venerealis* bv. intermedius	Argentina	1989	Bovine (vaginal mucus)	
01/165	GCA_001686885.1	*Campylobacter fetus* subsp. *venerealis* bv. intermedius	Argentina	2001	Bovine (vaginal mucus)	
03-293	GCA_000512745.2	*Campylobacter fetus* subsp. *venerealis* bv. intermedius	Argentina	2003	Bovine (fetus lung)	

1. Culture collection from the Department of Primary Industries in New South Wales, Australia. 2. Culture collection from the Institute for Glycomics at Griffith University in Queensland, Australia. 3. Culture collection from the Queensland Alliance for Agriculture and Food Innovation at the University of Queensland in Queensland, Australia.

### Oxford Nanopore long-read sequencing

The Ligation Sequencing Kit SQK-LSK-109 (Oxford Nanopore Technologies, Cambridge, UK) was used to prepare sequencing libraries from the double-stranded high molecular weight genomic DNA. The sequencing libraries were loaded onto MinION (Oxford Nanopore Technologies, Cambridge, UK) for long-read sequencing with MinKNOW software (Oxford Nanopore Technologies, Cambridge, UK). Approximately 1 Gbp of data were generated for each isolate, and modified base-calling from raw signal data with minimum quality score filtering of 8 was performed using Guppy 5.0.7.

### Illumina short-read sequencing

Extracted DNA was sent to the Ramaciotti Center for Genomics (University of New South Wales, Sydney, Australia) for short-read sequencing to generate 4 million read pairs or 1 Gbp of data. The libraries were prepared using the Nextera DNA Flex library prep kit (Illumina, California, USA), and the paired-end sequencing was executed on an iSeq 100 i1 sequencer with >80% bases higher than Q30 at 2 × 150 bp. The quality of the reads was assessed using FastQC 0.11.4 (Andrew, 2010) and was trimmed with Trimmomatic 0.39.1 using the paired-end mode (Bolger et al., 2014).

### Oxford Nanopore sequencing reads quality control and filtering

Porechop 0.2.4 (Wick et al., 2017a) was utilized to first remove the adapters, while NanoFilt 2.7.0 (De Coster et al., 2018) was implemented to select for reads that were >8,000 bp in length and >10 in quality score. The quality of the Nanopore long-read sequencing data was assessed and visualized using FastQC 0.11.4 (Andrew, 2010) and NanoPlot 1.3.0 (De Coster et al., 2018).

### Long-read assembly, assembly polish, assembly evaluation, and assembly annotation

Quality long reads were assembled into contigs using Trycycler 0.5.0 (Wick et al., 2017b). Briefly, the read files were divided into 12 subsets, with three subsets of each assembled using Flye 2.9 (Kolmogorov et al., 2019), Miniasm+Minipolish v0.1.3 (Vaser et al., 2017), Raven v1.5.1 (Vaser and Šikić, 2021), and Redbean v2.5 (Ruan and Li, 2020). The resulting long-read assemblies were grouped into per-replicon clusters. The cluster containing contigs with a genome size closest to the reference genome was manually selected for the reconciliation step, aiming to circularize the replicons.

Trycycler then conducted multiple sequence alignments of the contigs within each cluster and generated a consensus sequence for the final assembly. The expected genome size for each bacterial strain was determined based on their respective published reference genome available in the NCBI database (NCBI Resource Coordinators, 2018).

To polish the complete genomes derived from Nanopore long reads, Medaka 1.4.2 with model r941_min_high_g303 and Nanopolish 0.13.2 (Loman et al., 2015) were used. Genome polishing was accelerated using GNU Parallel (Tange, 2011). The draft assemblies were further refined by polishing with their corresponding Illumina short-read data using Pilon 1.24 (Walker et al., 2014).

The quality of the complete genomes was evaluated with Samtools 1.10 (Li et al., 2009) and QUAST 5.0.2 (Gurevich et al., 2013) using both long and short reads. The quality assessments generated for each polished assembly were combined and presented using MultiQC 1.10.1 (Ewels et al., 2016). The polished genomes were then visualized and validated using Artemis (Carver et al., 2011). The identities of the complete genomes were confirmed using BLAST (Altschul et al., 1990). Finally, the polished assemblies were submitted to the NCBI and annotated using the Prokaryotic Genome Annotation Pipeline (PGAP; Tatusova et al., 2016).

### Whole-genome comparison of *Campylobacter fetus* complete genomes

For a more comprehensive whole-genome comparison, other complete genomes of *C. fetus* subsp. *fetus* (*n* = 7) and *C. fetus* subsp. *venerealis* (*n* = 9) were downloaded from the NCBI Genome database (NCBI Resource Coordinators, 2018) (Table 1). The inconsistencies of different annotation tools employed in previous studies were taken into consideration. Therefore, the complete genome sequence fasta files of the 16 *C. fetus* strains sequenced in previous studies were reannotated using the same parameters as the nine *C. fetus* strains sequenced in this study. The whole-genome average nucleotide identity (ANI) of the 25 *C. fetus* subspecies genomes was computed using FastANI 1.33 using the all-against-all mode (Jain et al., 2018). The correlation of the 25 *C. fetus* subspecies' complete whole genomes based on their ANI was computed in R (R Core Team, 2018) using the “corrplot” package (Wei, 2021).

Prokka 1.14.6 (Seemann, 2014) was utilized to annotate the complete genome sequences (*n* = 25). Briefly, Prokka 1.14.6 (Seemann, 2014) identified the protein-coding regions using Prodigal 2.6.3 (Hyatt et al., 2010), followed by the functional annotation of the encoded protein by similarity search against protein databases. *Campylobacter fetus* subsp. *fetus* 04/554 (GCA_000759485.1) and *C. fetus* subsp. *venerealis* ATCC 19438^T^ (GCA_008271385.1) were provided as reference genomes for Prokka annotations to minimize the biases in annotation files for downstream analysis. The putative genomic islands (GIs) were predicted using IslandViewer 4 (Bertelli et al., 2017). The annotated assemblies were submitted to Roary 3.13.0 (Page et al., 2015) for pan-genome calculation. A heatmap was computed in R (R Core Team, 2018) using the “pheatmap” package (Kolde, 2019) to visualize the relationship of the 25 *C. fetus* subspecies based on the Roary results. The gene content and differences between the two closely related subspecies were also computed with GenAPI 1.0 (Gabrielaite and Marvig, 2019). The virulence factors known to be associated with *Campylobacter* were downloaded from the Virulence Factor Database (VFDB; Chen et al., 2005) for building a *Campylobacter*-specific VFDB database using ABRicate 1.0.1 (Seemann, 2016). The virulence factor encoding regions in each of the *C. fetus* subspecies were identified using the ABRicate 1.0.1 (Seemann, 2016) program.

SNPs were identified using Parsnp 1.5.6 (Treangen et al., 2014) from the whole-genome alignment generated with the complete genomes of the 25 *C. fetus* subspecies. A phylogenetic tree based on the SNPs identified in the whole-genome alignment of the 25 *C. fetus* strains was generated in R (R Core Team, 2018) using the “ape” package (Paradis and Schliep, 2018). The putative recombination regions with high SNP density were detected using Gubbins 3.0.0 (Croucher et al., 2014). The recombination-filtered SNPs were analyzed and annotated with SnpEff 4.3t (Cingolani et al., 2012) to filter out SNPs that potentially induce synonymous amino acid changes. The amino acid change was verified by examining the translated protein sequences of the SNP-coding coding sequences (CDS). The recombination-filtered synonymous SNPs that were different between *C. fetus* subsp. *fetus* and *C. fetus* subsp. *venerealis*, which are not present in putative GIs, were labeled as “candidate SNPs.” Additionally, SNPs that were different between *C. fetus* subsp. *venerealis* and its biovar intermedius variant were labeled as “biovar SNPs.” Any CDS that encoded for candidate SNPs were functionally annotated using eggNOG-mapper 2.1.6 (Cantalapiedra et al., 2021).

The annotated proteins of the SNP-coding CDS were retrieved from Prokka 1.14.6 (Seemann, 2014), and the interactions between the proteins were computed using STRING v11 (Szklarczyk et al., 2019) with *C. fetus* subsp. *venerealis* set as the organism of interest. The CDS, which were annotated with the “Peptidoglycan Biosynthesis” KEGG pathway, and their neighboring CDS with ≥8-degree functional association were taken for further investigation due to their potential association to the differential glycine tolerance among the subspecies. These SNPs were labeled as “Peptidoglycan SNPs.” To further verify the potential of the peptidoglycan SNPs for differentiation assay, an additional 33 curated and contamination-free RefSeq assemblies (13 *C. fetus* subsp. *fetus* and 20 *C. fetus* subsp. *venerealis* and its biovar) were downloaded from the NCBI Genome database (https://www.ncbi.nlm.nih.gov/datasets/genome) on 24th May 2024. The search terms “*Campylobacter fetus* subsp. *fetus*” and “*Campylobacter fetus* subsp. *venerealis*” were used, with the filter “Annotated by NCBI RefSeq” applied. The base change and amino acid change resulting from the peptidoglycan SNPs were verified across 58 *C. fetus* assemblies. The subspecies identification of each strain was compared with the phenotypic and molecular tests reported in previous studies.

Whole-genome alignment of the 25 *C. fetus* complete genomes was computed and visualized using the Blast Ring Image Generator (BRIG) 0.95 (Alikhan et al., 2011), which incorporated BLAST+ 2.10.1 (Camacho et al., 2009) for genome alignment. Additionally, the genes that were used in the published *C. fetus* subspecies identification PCR assays, including sodium/hydrogen exchanger family protein (*nahE*; Abril et al., 2007), *ISCfe1* (Abril et al., 2007), peptide transporter carbon starvation (*cstA*; Hum et al., 1997) and *parA* (Hum et al., 1997; McMillen et al., 2006) are downloaded from the NCBI nucleotide database (NCBI Resource Coordinators, 2018). The location of putative GIs, candidate SNPs, and existing PCR targets for *C. fetus* subspecies identification was identified and labeled on the alignment image generated using BRIG (Alikhan et al., 2011).

### Confirmation of *C. fetus* subspecies differentiation by TaqMan SNP quantitative PCR

One of the above-identified *C. fetus* subspecies SNPs (*mraY* gene SNP) was further exploited as a subspecies differentiating qPCR assay due to its potential association with the different glycine tolerance among *C.fetus* subspecies. Custom TaqMan MGB probes were designed using the Thermo Fisher Scientific online tool (Custom TaqMan™ SNP Genotyping Assay) targeting the *C. fetus* subsp. *venerealis* and *C. fetus* subsp. *fetus mraY* genes and labeled with VIC™ and FAM™ reporter dyes, respectively. *mraY* Forward primer: 5′ AAAATGATGATGAATTGGCGCCATT 3′; *mraY* Reverse primer: 5′ TGTGATGGAAACCTTATCTGTTATATTGCA 3′; *C. fetus* subsp. *fetus mraY* Probe: 5′FAM- CGTTTTTTGCGTATTTT-3′MGBNFQ; *C. fetus* subsp. *venerealis mraY* Probe: 5′VIC- CCGTTTTTTGTGTATTTT 3′MGBNFQ. The two probes and the forward and reverse primers were pre-mixed by Applied Biosystems and provided as a 20x mix for use in custom assays (Thermo Fisher Scientific, Australia). A 10 μL qPCR reaction was set up in duplicate using the AgPath-ID™ One-Step RT-PCR Reagent (Thermo Fisher Scientific) without the reverse transcriptase step consisting of 1x buffer, 900 nM of forward and reverse primers, 200 nM of the *C. fetus* subsp. *fetus* FAM probe, 200 nM of the *C. fetus* subsp. *venerealis* VIC probe, and 0.4 μl of the 25X RT-PCR Enzyme Mix (AmpliTaq Gold™ DNA Polymerase at 0.025 units per reaction). For positive controls, 2 ng of *C. fetus* subsp. *fetus* DNA (ATCC 27374) or 2 ng of *C. fetus* subsp. *venerealis* DNA (ATCC 19438) was added. A No Template Control (NTC, negative control) was included in every run. The assays were cycled in the Bio-Rad CFX96 Touch™ Real-Time PCR Detection System under the following conditions: activation at 95°C 10 min, followed by 45 cycles of 95°C 15 s, 69°C 1 min, and a final extension at 69°C for 7 min. Raw amplification data [quantification cycle (Cq) values and Relative Fluorescence Units (RFU)] were exported for analysis in Excel and R Studio (R Core Team, 2018) using the ggplot2 package (Wickham, 2016). The assay was screened against the following isolates: *C. fetus* subsp. *venerealis* ATCC19438 (positive control), A8, 957, 76223, 924, and 926; *C. fetus* subsp. *fetus* ATCC27374 (positive control) and BT376/03 (Supplementary Table 1). Other closely related bacterial species were also included as further controls: *Campylobacter hyointestinalis* strain 337, *Arcobacter cryaerophilus* strain 312, *Campylobacter ureolyticus* strain 412, and *Campylobacter sputorum* strain 530, which were isolated in a previous study screening abattoir bull penises and have been shown to cross-react in previous *C. fetus venerealis* molecular assays (McMillen et al., 2006; Spence et al., 2011).

## Results

### Whole-genome sequencing data, assembly, and annotations

The average read length, average N50, and average read quality of the nine strains sequenced with ONT were 33.52 kb, 38.43 kb, and 12.61, respectively (Supplementary Table 2). Illumina short-read sequencing yielded a total mean of 416,870 paired-end raw reads for each sample, which provided a mean coverage of 64X for each bacterial isolate (Supplementary Table 3).

All *C. fetus* strains were assembled into complete circular genomes. The average assembly size of *C. fetus*. *fetus* and *C. fetus* subsp. *venerealis* were 1,818,690 and 2,136,077 bp, respectively (Table 2). The QUAST quality assessment of each assembly showed that the long reads and short reads provided an average of 745.73X and 64.56X, respectively, to the assemblies (Supplementary Tables 4, 5). The average percentage of mapped long reads and mapped short reads were 98.56 and 97.50%, respectively. The complete genomes generated in this study and published genomes of *C. fetus* subsp. *fetus* and *C. fetus* subsp. *venerealis* were annotated with an average of 1,901 and 2,177 genes, respectively (Supplementary Table 6).

**Table 2:** Details of *Campylobacter fetus* complete genomes assembled in this study.

**Sample ID**	**Species**	**Assembly size (bp)**	**GC content (%)**
76223	*Campylobacter fetus* subsp. *venerealis*	2,105,546	33.47
924	*Campylobacter fetus* subsp. *venerealis* bv. intermedius	2,250,778	33.48
926	*Campylobacter fetus* subsp. *venerealis* bv. intermedius	2,123,600	33.48
957	*Campylobacter fetus* subsp. *venerealis*	2,088,026	33.41
A8	*Campylobacter fetus* subsp. *venerealis*	2,112,436	33.42
BT268/06	*Campylobacter fetus* subsp. *fetus*	1,909,714	33.26
BT376/03	*Campylobacter fetus* subsp. *fetus*	1,800,589	33.22
M20-08756/1A	*Campylobacter fetus* subsp. *fetus*	1,782,221	33.10
M20-04752/1B	*Campylobacter fetus* subsp. *fetus*	1,782,237	33.10

### Whole-genome comparison between 25 *Campylobacter fetus* strains

**Figure 1:**
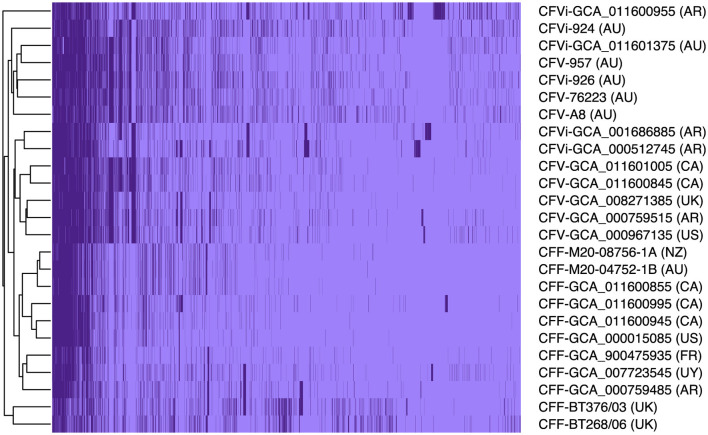
The pangenome of the 25 *Campylobacter fetus* strains. Dark purple indicates the presence of the gene ortholog, while light purple represents the absence of the gene ortholog. International Organization for Standardization (ISO) country code: Canada (CA), United States (US), France (FR), Uruguay (UY), Argentina (AR), United Kingdom (UK), Australia (AU), and New Zealand (NZ).

Pangenome analysis revealed that 1,561 core genes and 1,064 accessory genes were shared among the 25 *C. fetus* strains. The *C. fetus* subsp. *fetus* and *C. fetus* subsp. *venerealis* strains were separated in the hierarchical tree generated based on the presence and absence of gene orthologs (Figure 1). The pangenome analysis demonstrated the gene ortholog, which encoded for a peptidase S24 LexA-like protein, was exclusively encoded in all the *C. fetus* subsp. *venerealis* genomes but not in any of the *C. fetus* subsp. *fetus* genomes. However, three paralogs also encoded for peptidase S24 LexA-like proteins and were predicted in some of the *C. fetus* subsp. *fetus* and *C. fetus* subsp. *venerealis* strains (Supplementary Table 7).

In contrast, *C. fetus* subsp. *venerealis* and the bv. intermedius variant did not fall into distinct branches because none of the gene orthologs was able to differentiate *C. fetus* subsp. *venerealis* and *C. fetus* subsp. *venerealis* bv. intermedius. Interestingly, the six Australian *C. fetus* subsp. *venerealis* strains (GCA_011601375, A8, 924, 926, 957, and 76223) formed a separated clade from the other non-Australian *C. fetus* subsp. *venerealis*. A total of 14 gene orthologs were identified in the Australian *C. fetus* subsp. *venerealis* strains but not in the non-Australian strains, of which one was present in all *C. fetus* subsp. *fetus*, and nine were absent in all the *C. fetus* subsp. *fetus* strains. On the other hand, 37 gene orthologs were present in the non-Australian *C. fetus* subsp. *venerealis* and not the Australian strains, none of which were identified in all *C. fetus* subsp. *fetus*, and 34 were absent in all the *C. fetus* subsp. *fetus* strains.

**Figure 2:**
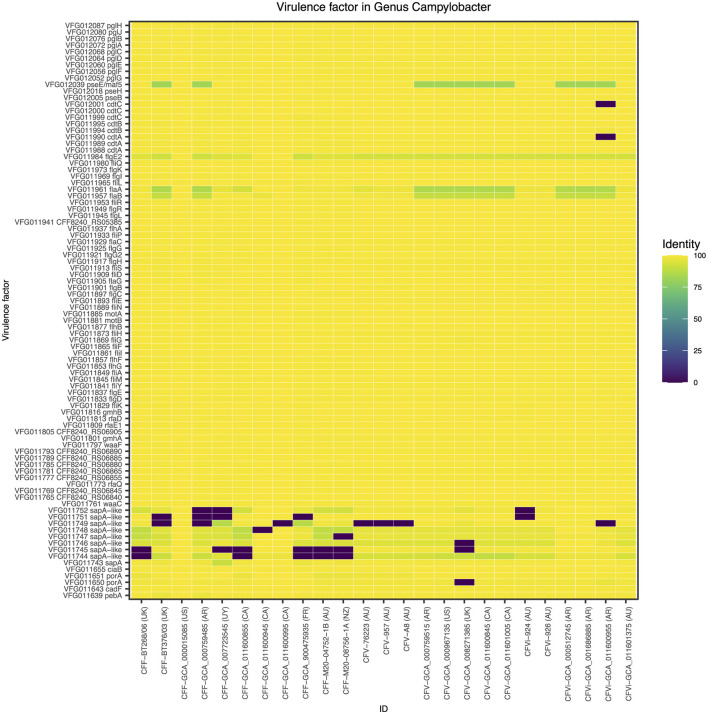
The *Campylobacter*-specific virulence factors among the 25 *Campylobacter fetus* strains. The color key indicates the percentage of identity between the genomic regions identified in each isolate and the *Campylobacter*-specific virulence factors, in which yellow represents 100% identity and purple represents 0% identity. International Organization for Standardization (ISO) country code: Canada (CA), United States (US), France (FR), Uruguay (UY), Argentina (AR), United Kingdom (UK), Australia (AU), and New Zealand (NZ).

A closer look at the genomic regions encoding the *Campylobacter*-specific virulence factors demonstrated that the 25 *C. fetus* subspecies commonly expressed genomic regions encoding 88 virulence factors (Figure 2). Nine virulence factors were not expressed in some of the *C. fetus* subspecies but not explicitly in either *C. fetus* subsp. *fetus* or *C. fetus* subsp. *venerealis*. The highest variation was observed within the S-layer proteins, which are categorized under the class of “colonization and immune evasion” in VFDB. Nonetheless, the variations among the predicted proteins were not consistent within either of the subspecies or the biovar.

**Figure 3:**
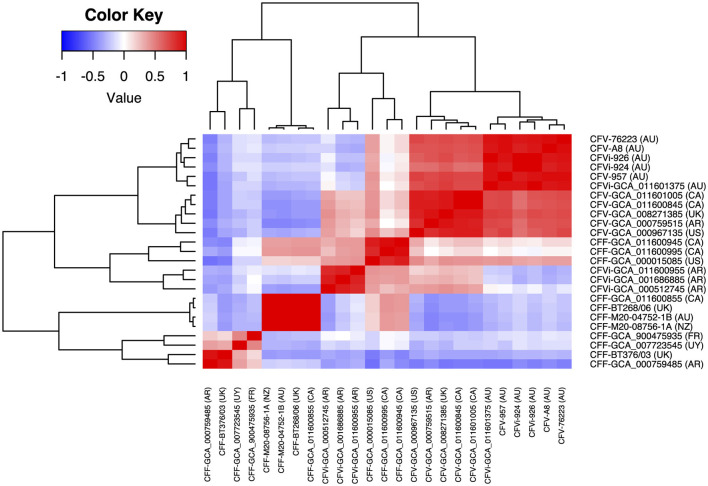
Correlation between the 25 *Campylobacter fetus* strains based on their average nucleotide identity. The color key indicates the degree of correlation between the strains, in which red represents the highest correlation while blue represents the lowest correlation. The dendrogram demonstrates the hierarchical relationship between the 25 *Campylobacter fetus* strains based on their average nucleotide identity. International Organization for Standardization (ISO) country code: Canada (CA), United States (US), France (FR), Uruguay (UY), Argentina (AR), United Kingdom (UK), Australia (AU), and New Zealand (NZ).

The average nucleotide identity between 25 *C. fetus* strains was more than 95%. The correlation tree based on the ANI showed that eight of the *C. fetus* subsp. *fetus* strains, including GCA_011600855, BT268/06, M20-04752/1B, M20-08756/1A, GCA_900475935^T^, GCA_007723545, BT376/03, and GCA_00759485, formed a separate branch from the *C. fetus* subsp. *venerealis* strains (Figure 3). The other three *C. fetus* subsp. *fetus* strains (GCA_000015085, GCA_011600995, and GCA_011600945) shared the same ancestor with three *C. fetus* subsp. *venerealis* bv. intermedius strains (GCA_011600955, GCA_001686885, and GCA_000512745) from Argentina. The remaining *C. fetus* subsp. *venerealis* strains (*n* = 11) formed a separate clade, in which the *C. fetus* subsp. *venerealis*, including both biovars from Australia (GCA_011601375, A8, 924, 926, 957, and 76223) clustered separately to all other *C. fetus* subsp. *venerealis* from around the world.

**Figure 4:**
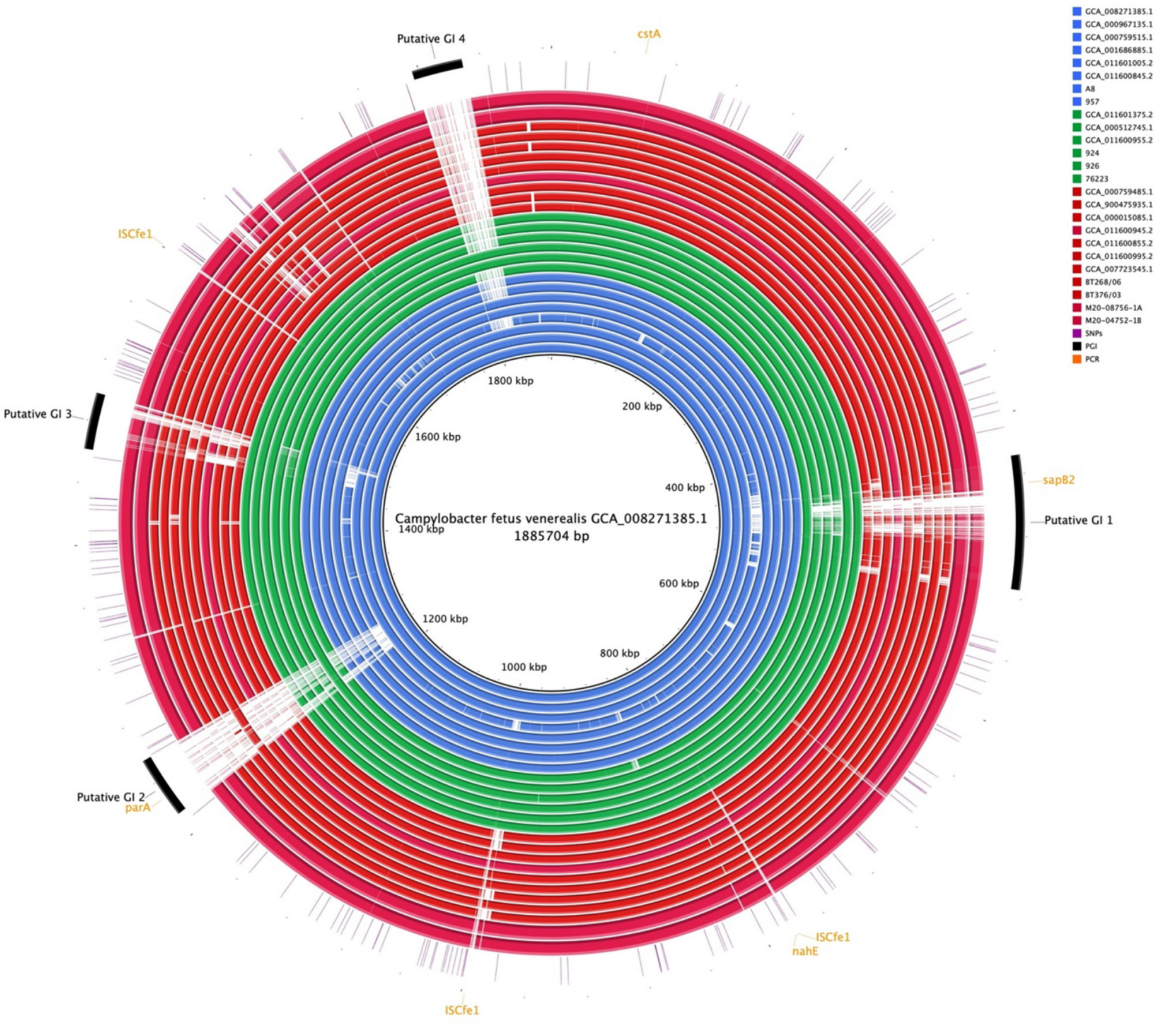
Whole-genome alignment of 25 *Campylobacter fetus* subspecies. Reference: published *C. fetus* subsp. *venerealis* ATCC 19438^T^ [GCA_008271385.1]. Query were complete genome sequences of *C. fetus* subsp. *Fetus* (*n* = 11) and *C. fetus* subsp. *venerealis* (*n* = 14) subspecies. Strains labeled in red are *C. fetus* subsp. *fetus*, isolated and labeled in blue, are *C. fetus* subsp. *venerealis* and strains labeled in green are *C. fetus* subsp. *venerealis* bv. intermedius. Black arcs represent the putative genomic islands. Orange arcs represent the genes that were used in PCR *C. fetus* subspecies identification. Purple lines represent candidate SNPs identified in the 25 *C. fetus* subspecies.

Comparative genome alignment of the *C. fetus* strains (*n* = 25) illustrated the high level of genome synteny between the *C. fetus* subspecies (Figure 4). Missing genomic regions were inconsistently observed among the 25 *C. fetus* strains where putative GIs were predicted. PCR target genes, including *sapB2* and *par*A, were located on putative GIs.

**Figure 5:**
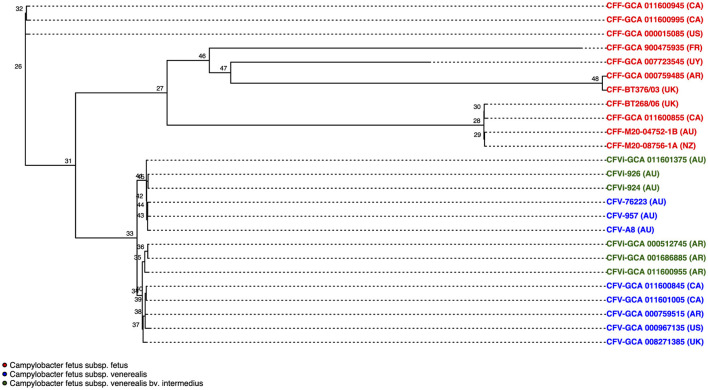
Phylogenetic tree based on single nucleotide polymorphisms (SNPs) between the 25 *Campylobacter fetus* strains. Strains labeled in red are *C. fetus* subsp. *fetus*, strains labeled in blue are *C. fetus* subsp. *venerealis*, and strains labeled in green are *C. fetus* subsp. *venerealis* bv. intermedius. All *C. fetus* subsp. *fetus* isolates clustered into a separate branch from all *C. fetus* subsp. *venerealis* and *C. fetus* subsp. *venerealis* bv. intermedius isolates, indicating that there were distinctive SNPs. *C. fetus* subsp. *venerealis* and *C. fetus* subsp. *venerealis* bv. intermedius isolates from Australia clustered separately from non-Australian isolates. International Organization for Standardization (ISO) country code: Canada (CA), United States (US), France (FR), Uruguay (UY), Argentina (AR), United Kingdom (UK), Australia (AU), and New Zealand (NZ).

The phylogenetic tree that resulted from the whole-genome alignment clustered *C. fetus* subsp. *venerealis* into a separate branch from the *C. fetus* subsp. *fetus* (Figure 5). Moreover, the phylogenetic tree also clustered the Australian *C. fetus* subsp. *venerealis* in a separate branch from the *C. fetus* subsp. *venerealis* identified in the United Kingdom, United States, Canada, and Argentina (Figure 5). Both within the Australian and non-Australian *C. fetus* subsp. *venerealis* clades, the *C. fetus* subsp. *venerealis* bv. intermedius were clustered separately from the normal *venerealis* variant.

**Figure 6:**
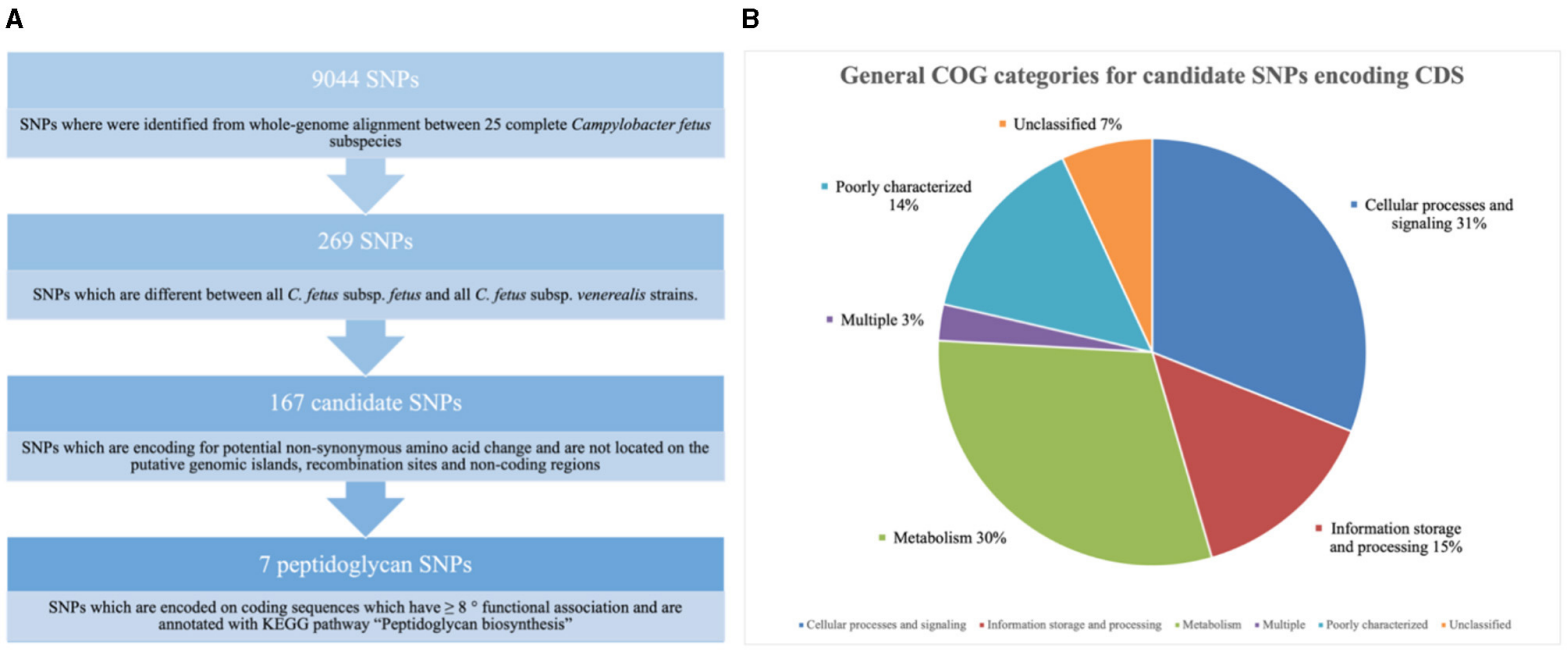
**(A)** Screening for single nucleotide polymorphisms (SNPs) suitable for reliable differentiation between *Campylobacter fetus* subsp. *fetus* and *Campylobacter fetus* subsp. *venerealis*. **(B)** Candidate single nucleotide polymorphisms (SNPs) are categorized into Clusters of Orthologous Groups (COGs). The blue section represents coding sequences (CDS) responsible for “cellular processes and signaling” (*n* = 45). The orange section represents the CDS responsible for “information storage and processing” (*n* = 21). The gray section represents CDS responsible for “metabolism” (*n* = 44). The yellow section represents CDS belonging to multiple groups of COGs (*n* = 4). The light blue section represents CDS, which was poorly categorized (*n* = 21). The green section represents CDS with no match return from querying the COG database (*n* = 10).

Nine thousand and 44 SNPs were identified from the core genome, of which only 269 SNPs were different between all *C. fetus* subsp. *fetus* and all *C. fetus* subsp. *venerealis* strains (Figure 6A). The SNPs that contributed to putative synonymous amino acid change, located on putative GIs, and involved in recombination events were filtered out from downstream analysis. The remaining 184 SNPs were labeled as “candidate SNPs,” of which 17 were located in non-coding regions, and 167 were located in CDS (Supplementary Table 8). A total of 16 of the CDSs were encoded for two candidate SNPs, with one CDS encoded for three candidate SNPs and the other CDS encoded for five candidate SNPs. In total, the 167 candidate SNPs were located on 145 CDS, of which 15 of the CDS were encoding for hypothetical proteins. The majority of the SNPs-encoding CDS were responsible for “cellular processes and signaling” (*n* = 45), followed by “metabolism” (*n* = 44) and “information storage and processing” (*n* = 21; Figure 6B, Supplementary Table 9).

**Figure 7:**
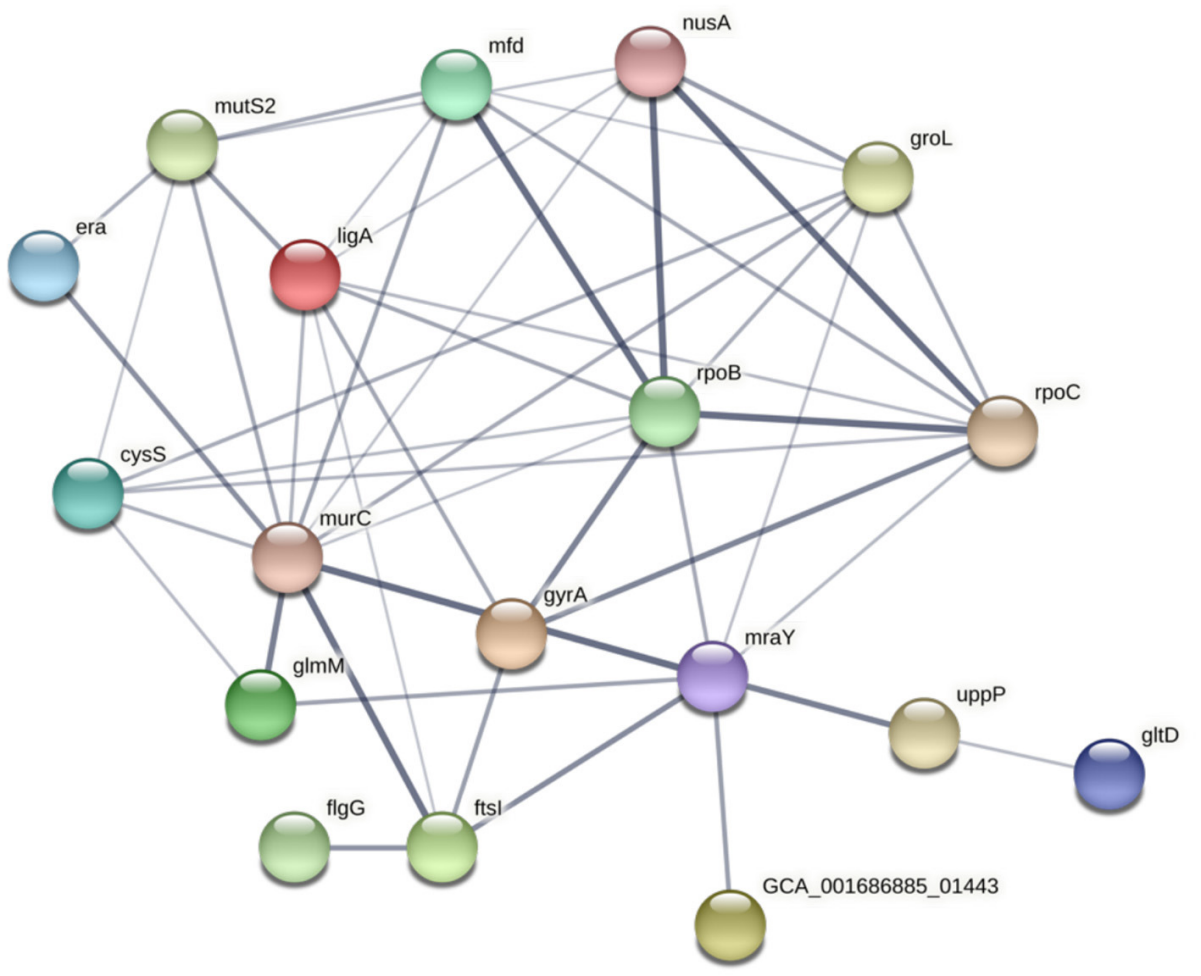
STRING network of SNP-coding CDS, which had more than eight degrees of association. The thickness of the network edges indicates the confidence level of the functional interactions.

The potential functional association between the SNP-coding CDS was determined, and 41 candidate SNPs were identified on the SNP-coding CDS, which posed more than eight degrees of association. A subset of the 41 SNPs that were annotated with the KEGG pathway “Peptidoglycan biosynthesis” (Supplementary Figure 1) was chosen for further analysis due to their potential association with the differential glycine production in the *C. fetus* subspecies. The subset included *murC, ftsI, uppP*, and *mraY*, and their first neighbors (Figure 7). Among them, the SNP-coding CDS encoded for the candidate SNPs associated with significant amino acid change, including *rpoC, cysS, rpoB, flgG, mfd, mraY*, and *mutS2*, were manually verified (Table 3). The candidate SNPs in these SNP-coding CDS were referred to as peptidoglycan SNPs (Figure 6A).

**Table 3:** Amino acid and DNA base changes on the candidate SNP-coding CDS encoded on *C. fetus* subsp. *fetus* and *C. fetus* subsp. *venerealis*.

**Gene**	**Product**	**Position on GCA_008271385.1**	**Locus tag on GCA_008271385.1**	**Type**	***Campylobacter fetus*** **subsp**. ***fetus***		***Campylobacter fetus*** **subsp**. ***venerealis***	
					**Amino acid**	**DNA base**	**Amino acid**	**DNA base**
*cysS*	cysteinyl-tRNA	799900	CFVT_0790	Missense	Cys	C	Tyr	T
*flgG*	flagellar	549585	CFVT_0554	Missense	Asn	A	Ser	G
*mfd*	transcription-repair	744016	CFVT_0740	Missense	Thr	C	Ile	T
*mraY*	phospho-N-acetylmuramoyl-pentapeptide transferase	1384375	CFVT_1392	Upstream variant	Arg	C	His	T
*mutS2*	Endonuclease	653990	CFVT_0655	Missense	Ala	C	Val	T
*rpoB*	DNA-dependent RNA polymerase	1351503	CFVT_1350	Missense	Asp	C	Asn	T
*rpoC*	DNA-dependent RNA polymerase	1349348	CFVT_1349	Missense	Leu	G	Phe	A

Interestingly, there were no common SNPs that could absolutely separate all the *C. fetus* subsp. *venerealis* and its biovar intermedius variant regardless of the country of origin. Therefore, this study only tested the potential of the seven peptidoglycan SNPs in differentiating the *C. fetus* subsp. *fetus* (CFF) from *C. fetus* subsp. *venerealis* and its biovar (CFV/CFVi). On top of the 25 complete genomes, an additional 33 curated RefSeq assemblies (13 *C. fetus* subsp. *fetus* and 20 *C. fetus* subsp. *venerealis* and its biovar) were recruited in the evaluation (Table 4). All seven peptidoglycan SNPs performed consistently and reliably divided the assemblies into two groups (Supplementary Table 10). The 24 *C. fetus* subsp. *fetus* assemblies, except NWU_ED24 (GCF_013406925.1), 13/344 (GCF_008527615.1), and 08/421 (GCF_008526335.1), posed SNP pattern conformed to the CFF subset. Interestingly, the three assemblies (NWU_ED24, 13/344, and 08/421) posed an SNP pattern that conformed to the CFV/CFVi subset. On the other hand, the 34 *C. fetus* subsp. *venerealis* assemblies, except P4531 (GCF_016406645.1), posed an SNP pattern conformed to the CFV/CFVi subset. The P4531 strain posed SNP pattern conformed to the CFF subset instead. Therefore, in this study, NWU_ED24, 13/344, and 08/421 were classified as *C. fetus* subsp. *venerealis* and its biovar, while the P4531 strain was classified as *C. fetus* subsp. *fetus*.

**Table 4:** Characterization of 58 *Campylobacter fetus* subspecies according to previous studies and the current study.

**Assembly accession**	**Strain**	**Assembly level**	**NCBI annotation**	^*^Identification #1	^*^Identification #2	^*^Identification #3	^*^Identification #4	**Identification in this study**
CP075536-CP075537	A8	Complete	CFV					CFV/CFVi
GCF_030544605.1	CFV924	Complete	CFVi					CFV/CFVi
GCF_030544585.1	CFV926	Complete	CFVi					CFV/CFVi
GCF_030544565.1	CFV957	Complete	CFV					CFV/CFVi
GCF_030544545.1	CFV76223	Complete	CFV					CFV/CFVi
GCF_016612985.1	SA21-221439	Contig	CFV	CFV (Silva et al., 2021)				CFV/CFVi
GCF_016612955.1	SA21-217832	Contig	CFV	CFV (Silva et al., 2021)				CFV/CFVi
GCF_016612945.1	SA21-217833	Contig	CFV	CFV (Silva et al., 2021)				CFV/CFVi
GCF_016612925.1	IS16-01257	Contig	CFV	CFV (Silva et al., 2021)				CFV/CFVi
GCF_016612875.1	IS26-07793	Contig	CFV	CFV (Silva et al., 2021)				CFV/CFVi
GCF_016406645.1	P4531	Complete	CFV	CFV (Kim et al., 2021)				CFF
GCF_013406955.1	NWU_ED23	Contig	CFV	CFV (Tshipamba et al., 2020a)	CFVi (Silva et al., 2021)	CFF/CFVi (Farace et al., 2021)		CFV/CFVi
GCF_012274465.1	NW_ME2	Contig	CFV	CFV (Tshipamba et al., 2020b)	CFVi (Silva et al., 2021)			CFV/CFVi
GCF_008526355.1	06/341	Contig	CFV	CFV (Hum et al., 1997)	CFVi (Farace et al., 2021, 2022)	CFF/CFVi (Farace et al., 2019)		CFV/CFVi
GCF_008271385.1	NCTC 10354	Complete	CFV	CFV (van Bergen et al., 2005a; Farace et al., 2019)				CFV/CFVi
GCF_002592365.1	66Y	Contig	CFV	CFV (Farace et al., 2019; Iraola et al., 2017)				CFV/CFVi
GCF_002592335.1	TD	Contig	CFV	CFV (Farace et al., 2019; Iraola et al., 2017)				CFV/CFVi
GCF_001699745.1	ADRI513	Contig	CFVi	CFVi (van der Graaf-van Bloois et al., 2016a)	CFV/CFVi (van der Graaf-van Bloois et al., 2014)	CFF/CFVi (Farace et al., 2019)		CFV/CFVi
GCF_001699735.1	zaf3	Contig	CFVi	CFVi (van der Graaf-van Bloois et al., 2014)	CFF/CFVi (Farace et al., 2019)			CFV/CFVi
GCF_001699685.1	cfvi9825	Contig	CFVi	CFVi (van der Graaf-van Bloois et al., 2014; van Bergen et al., 2005b)	CFV (Farace et al., 2019)			CFV/CFVi
GCF_001699645.1	CCUG 33872	Contig	CFV	CFV (Willoughby et al., 2005)	CFVi (van der Graaf-van Bloois et al., 2016a)	CFF/CFVi (Farace et al., 2019)	CFV/CFVi (van der Graaf-van Bloois et al., 2014)	CFV/CFVi
GCF_001699615.1	cfvi03/596	Contig	CFVi	CFVi (van der Graaf-van Bloois et al., 2014, 2016a; Farace et al., 2021)	CFF (Hum et al., 1997; Iraola et al., 2017)	CFF/CFVi (Farace et al., 2019)		CFV/CFVi
GCF_001699565.1	cfvi92/203	Contig	CFVi	CFVi (van der Graaf-van Bloois et al., 2014, 2016a)	CFV (Hum et al., 1997)	CFF/CFVi (Farace et al., 2019)		CFV/CFVi
GCF_001686885.1	01/165	Complete	CFV	CFVi (van der Graaf-van Bloois et al., 2014, 2016a; Farace et al., 2021; Iraola et al., 2017)	CFF (Hum et al., 1997)	CFF/CFVi (Farace et al., 2019)		CFV/CFVi
GCF_000967135.1	84-112	Complete	CFV	CFV (van der Graaf-van Bloois et al., 2014, 2016a; van Bergen et al., 2005a; Hum et al., 1997; Farace et al., 2019; Iraola et al., 2017)				CFV/CFVi
GCF_000759515.1	97/608	Complete	CFV	CFV (van der Graaf-van Bloois et al., 2014, 2016a; Hum et al., 1997; Farace et al., 2019, 2021; Iraola et al., 2017)				CFV/CFVi
GCF_000744035.1	B6	Scaffold	CFV	CFV (van der Graaf-van Bloois et al., 2016a; Farace et al., 2019; Iraola et al., 2017; Barrero et al., 2014)				CFV/CFVi
GCF_000744025.1	642-21	Scaffold	CFVi	CFVi (van der Graaf-van Bloois et al., 2016a; Barrero et al., 2014)	CFF/CFVi (Farace et al., 2019)			CFV/CFVi
GCF_000512745.2	cfvi03/293	Complete	CFVi	CFVi (Iraola et al., 2017)	CFV (Hum et al., 1997)	CFF/CFVi (van der Graaf-van Bloois et al., 2014, 2016a; Farace et al., 2019)		CFV/CFVi
GCF_000414135.1	99541	Contig	CFVi	CFVi (van der Graaf-van Bloois et al., 2016a; Iraola et al., 2013; Farace et al., 2021; Iraola et al., 2017)	CFF (Hum et al., 1997)	CFF/CFVi (Farace et al., 2019)		CFV/CFVi
GCA_011600845.2	08A1102-42A	Complete	CFV	CFV (Nadin-Davis et al., 2021; Farace et al., 2021; Mukhtar, 2013)				CFV/CFVi
GCA_011600955.2	ADRI1362	Complete	CFVi	CFVi (Nadin-Davis et al., 2021)	Cff/CFVi (van der Graaf-van Bloois et al., 2014, 2016a; Farace et al., 2019)			CFV/CFVi
GCA_011601005.2	08A948-2A	Complete	CFV	CFV (Nadin-Davis et al., 2021; Farace et al., 2021; Mukhtar, 2013)				CFV/CFVi
GCA_011601375.2	ADRI545	Complete	CFVi	CFVi (Nadin-Davis et al., 2021)				CFV/CFVi
GCF_000015085.1	82-40	Complete	CFF	CFF (van der Graaf-van Bloois et al., 2014, 2016a; van Bergen et al., 2005a; Iraola et al., 2017)	CFF/CFVi (Farace et al., 2019)			CFF
GCF_000759485.1	04/554	Complete	CFF	CFF (van der Graaf-van Bloois et al., 2014, 2016a; Hum et al., 1997; Farace et al., 2021; Iraola et al., 2017)	CFF/CFVi (Farace et al., 2019)			CFF
GCF_001399955.1	H1-UY	Contig	CFF	CFF (van der Graaf-van Bloois et al., 2016a; Iraola et al., 2015, 2017)	CFF/CFVi (Farace et al., 2019)			CFF
GCF_001699505.1	98/v445	Contig	CFF	CFF (van der Graaf-van Bloois et al., 2014, 2016a; van Bergen et al., 2005a)	CFV (Hum et al., 1997)	CFF/CFVi (Farace et al., 2019)		CFF
GCF_001699575.1	BT 10/98	Contig	CFF	CFF (van der Graaf-van Bloois et al., 2014, 2016a)	CFF/CFVi (Farace et al., 2019)			CFF
GCF_003426005.1	HC1	Contig	CFF	CFF (Iraola et al., 2017)	CFF/CFVi (Farace et al., 2019)			CFF
GCF_003426015.1	HC2	Contig	CFF	CFF (Iraola et al., 2017)	CFF/CFVi (Farace et al., 2019)			CFF
GCF_007723545.1	INIA/17144	Complete	CFF	CFF/CFVi (Farace et al., 2021)				CFF
GCF_008014295.1	D0052	Contig	CFF	CFF/CFVi (Farace et al., 2021)				CFF
GCF_008526335.1	08/421	Contig	CFF	CFF (Farace et al., 2021, 2022)	CFV (Hum et al., 1997)	CFF/CFVi (Farace et al., 2019)		CFV/CFVi
GCF_008527615.1	13/344	Contig	CFF	CFF (Hum et al., 1997; Farace et al., 2021, 2022)	CFF/CFVi (Farace et al., 2019)			CFV/CFVi
GCF_008693125.1	CCUG 6823 AT	Contig	CFF	CFF	CFF/CFVi (Farace et al., 2021)			CFF
GCF_013406925.1	NWU_ED24	Contig	CFF	CFF	CFF/CFVi (Farace et al., 2021)			CFV/CFVi
GCF_017896385.1	CITCf01	Complete	CFF	CFF (Lynch et al., 2021)				CFF
GCF_017896405.1	CITCf02	Complete	CFF	CFF (Lynch et al., 2021)				CFF
GCF_020828935.1	YZU0709	Contig	CFF	CFF (Li et al., 2022)				CFF
GCF_900475935.1	NCTC10842	Complete	CFF	CFF (van Bergen et al., 2005a; Farace et al., 2019; Willoughby et al., 2005)	CFF/CFVi (Farace et al., 2019)			CFF
GCA_011600855.2	02A725-35A	Complete	CFF	CFF (Nadin-Davis et al., 2021)				CFF
GCA_011600945.2	00A031	Complete	CFF	CFF (Nadin-Davis et al., 2021)				CFF
GCA_011600995.2	09A980	Complete	CFF	CFF (Nadin-Davis et al., 2021)				CFF
GCF_032594895.1	M20-08756/1A	Complete	CFF	CFF				CFF
GCA_030544625.1	BT376/03	Complete	CFF	CFF				CFF
GCA_030544645.1	BT268/06	Complete	CFF	CFF				CFF
GCF_032594815.1	M20-04752/1B	Complete	CFF	CFF				CFF

^*^The identification of the Campylobacter strain in the previous studies.

### Confirmation of *C. fetus* subspecies differentiation by TaqMan SNP quantitative PCR

**Figure 8:**
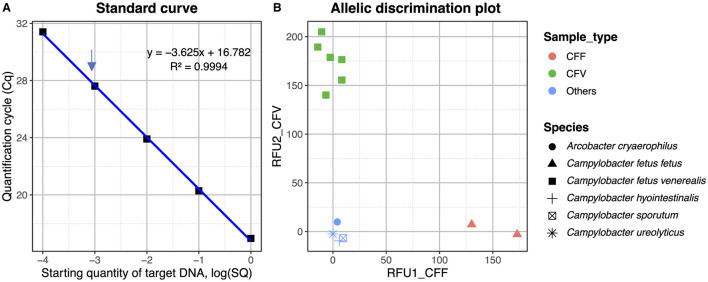
**(A)** Standard curve for the *mraY* SNP assay plotting the quantification cycle (Cq) value against the starting quantity of *C. fetus venerealis* DNA. Data points are from 1 ng to 0.1 pg, with 1 pg indicated by an arrow. The *R*^2^ of the standard curve is 0.994. **(B)** Allelic discrimination plot for *mraY* Taqman SNP assay using Relative Fluorescent Units (RFU) for VIC and FAM. Red—CFF control (ATCC 27374), brown—CFF isolate (strain BT376/03), green—CFV control (ATCC 19438), blue—CFV isolates (strains A8, 957, 76223, 924, and 926), and light blue—other *Campylobacter* species (*C. sputorum, C. ureolyticus, C. hyointestinalis*, and *A. cryoaerophilus*).

One of the SNPs differentiating the *C. fetus* subspecies located in the *mraY* gene (Table 3) was further exploited in a TaqMan SNP qPCR assay where the FAM channel detected the *C. fetus* subsp. *fetus* and the VIC channel *C. fetus* subsp. *venerealis*. The sensitivity of the assay was 1 pg when using pure bacterial DNA, and following testing of mixed *Campylobacter*-like species, a positive cut-off was determined at Cq 33 cycles (Figure 8A). The mraY assay was able to distinguish between *C. fetus* subsp. *venerealis* and *C. fetus* subsp. *fetus* controls, as well as other *C. fetus* isolates listed in Table 1 and other closely related *Campylobacter* species (*C. sputorum, C. ureolyticus, C. hyointestinalis*, and *A. cryoaerophilus*; Figure 8B).

## Discussion

Differentiating C. fetus subspecies is crucial for the routine screening of ruminants for import and export and epidemiological investigation. Subspecies differentiation has been investigated using multiple methods, including biochemical analysis (OIE, 2019), AFLP (Wagenaar et al., 2001), PFGE (On and Harrington, 2001), MLST (van Bergen et al., 2005b; Iraola et al., 2015), PCR (Hum et al., 1997; Abril et al., 2007; Wang et al., 2002), and genome comparison methods (van der Graaf-van Bloois et al., 2014; Kienesberger et al., 2014). The results of this study demonstrated an improved and less laborious genome analysis method that successfully separated *C. fetus* subsp. *fetus* and *C. fetus* subsp. *venerealis* into two distinct clusters. According to their phenotypes, similar to previous AFLP (Wagenaar et al., 2001) and PFGE analyses (On and Harrington, 2001), but with a more efficient procedure.

In this study, the Australian *C. fetus* subsp. *venerealis* were separated from the non-Australian *C. fetus* subsp. *venerealis*, which was different from the indistinguishable geographical genotypes described in the previous core genome analysis (van der Graaf-van Bloois et al., 2014) and PFGE analysis (On and Harrington, 2001). Unique microbial lineages due to geographical separation are not novel. For example, most Australian *Glaesserella* (*Haemophilus) parasuis* strains were found to belong to unique sequence types not previously recorded in the relevant MLST database (Turni et al., 2018). In the phylogenetic tree generated using whole-genome alignment, the *C. fetus* subsp. *venerealis* clade shared the same ancestor with eight other *C. fetus* subsp. *fetus* strains, potentially indicating that *C. fetus* subsp. *venerealis* was derived from *C. fetus* subsp. *fetus*. The identification of a small number of SNPs (*n* = 184), which are differentially expressed in the *C. fetus* subsp. *fetus* and *C. fetus* subsp. *venerealis* strains, which were supported by the observations from the MLST analyses, indicating a limited genetic variation between *C. fetus* subspecies was driven mainly by the slow accumulation of point mutations (van Bergen et al., 2005b). Additionally, this study did not identify any SNPs that are unique to the *C. fetus* subsp. *venerealis* bv. intermedius regardless of geographical origins, suggesting that clonal evolution occurred separately within the geographically different *C. fetus* subsp. *venerealis* clades. It appears that *C. fetus* subsp. *venerealis* and its biovar-acquired point mutations, which have been vertically transmitted and enabled the development of their highly niche-specific and pathogenicity-specific characteristics.

For the comparative whole-genome analysis, only complete *C. fetus* genomes were used. This approach ensured a comprehensive comparison and prevented false positives that could result from aligning the complete genome against the gaps of an incomplete genome. The previous investigation, compared one complete genome of *C. fetus* subsp. *fetus* (GCA_000015085) against one incomplete genome of *C. fetus* subsp. *venerealis* (GCA_000222425) reported that 88 and 428 gene families were unique to *C. fetus* subsp. *fetus* and *C. fetus* subsp. *venerealis*, respectively (Ali et al., 2012). The current study only identified one gene ortholog, which was different between the two complete genomes of the *C. fetus* subspecies. Our results aligned with the previous suggestion, based on methods other than genome sequencing, that there is a lack of genetic diversity between the *C. fetus* subspecies because the *C. fetus* strains are at an early evolutionary stage (van Bergen et al., 2005a; Wagenaar et al., 2001). The only unique gene ortholog encoded for a peptidase S24 LexA-like protein was only identified in *C. fetus* subsp. *venerealis* and not in the *C. fetus* subsp. *fetus* strains. However, three other paralogs coding for peptidase S24 LexA-like protein were identified in both *C. fetus* subspecies in this study. While LexA is a global transcription factor responsible for regulating host SOS response, several studies have also suggested that mobile genetic elements utilize the LexA activity of the hosts for their induction (Fornelos et al., 2016; Quinones et al., 2005; Kimsey and Waldor, 2009). The previous comparison of 14 *C. fetus* strains observed that the *lexA* gene, which served as a prophage regulator, was mostly identified at the boundary of a prophage element (Nadin-Davis et al., 2021).

Additionally, we found no gene ortholog that was unique to either *C. fetus* subsp. *venerealis* or the *C. fetus* subsp. *venerealis* biovar variant. This is in contrast to the previous pangenome analysis of 31 *C. fetus* subsp. *venerealis* strains using a mixture of complete and incomplete genomes, which identified inconsistent expressions of *parA* and T4SS encoding genes (*virB2-virB11* and *virD4*) in the *C. fetus* strains (Silva et al., 2021). Additionally, the virulence gene investigation in this study demonstrated variations in the *sap* genes among the 25 *C. fetus* strains. This result corresponded to the previous studies, which categorized *C. fetus* subspecies to serovars based on the variable expression of genes in the *sap* locus that encode for surface layer protein (SLP; Tu et al., 2001; Dworkin et al., 1995). Our results reinforced that the serovar classification is not unique at the *C. fetus* subspecies level.

The biochemical tests that are currently recommended by OIE (2019) for identifying *C. fetus* subspecies include glycine tolerance test and H_2_S production. *C. fetus* subsp. *fetus* is positive for both tests, while *C. fetus* subsp. *venerealis* is negative for both. Previous studies have suggested the ATP-binding cassette-type L-cysteine transporter as a potential marker for H_2_S-positive *C. fetus* strains, leading to the development of an accurate diagnostic test based on the L-Cys transporter-deletion polymorphism (van der Graaf-van Bloois et al., 2016a; Farace et al., 2019).

However, unlike *C. fetus* subsp. *venerealis*, the intermedius biovar variant is positive for H_2_S production. As a result, previous tests using molecular techniques to detect the L-Cys transporter-deletion polymorphism were unable to distinguish *C. fetus* subsp. *venerealis* biovar intermedius strains from *C. fetus* (Farace et al., 2019, 2021). This study provided 167 SNPs as new candidates for *C. fetus* subspecies genotyping, which may be valuable for precise and efficient routine screening on farms, international trade, and for epidemiological investigations and diagnostics.

In this study, SNP-coding CDS was investigated for a correlation with the differential tolerance to glycine among the *C. fetus* subspecies, which separates the *C. fetus* subsp. *fetus* from the *C. fetus* subsp. *venerealis* and its biovar intermedius. Glycine has been suggested to inhibit bacterial cell wall biosynthesis, particularly the peptidoglycan component, and thus can show an antibacterial effect (Hishinuma et al., 1969). Therefore, candidate CDS annotated with the “peptidoglycan biosynthesis” KEGG pathway and posed potential functional association with one another, including *cysS, flgG, mfd, mraY, mutS2, rpoB*, and *rpoC*, were recruited for further analysis. Gene *mraY* was part of the gene set found in peptidoglycan-intermediate obligate intracellular bacteria (Otten et al., 2018). Phospho-N-acetylmuramoyl-pentapeptide-transferase (*mraY*) is a catalytic enzyme that initiates the lipid cycle reactions during bacterial peptidoglycan synthesis (Struve et al., 1966). Under normal circumstances, *mraY* has high specificity to L-alanine and D-alanine (Hammes and Neuhaus, 1974). However, the non-synonymous amino acid change introduced by the peptidoglycan SNP identified in our study potentially modified *mraY* and thus allowed glycine substitution. As a result, bacteriolysis or morphological aberrations of the bacterial cells could be induced and lead to differential glycine tolerance between the *C. fetus* subspecies. The presence of this SNP was further exploited in a novel *C. fetus* subspecies TaqMan SNP qPCR, which demonstrated specific detection of each subspecies using different fluorophores and no detection of other closely related species such as *C. hyointestinalis* as reported for other diagnostic targets present on mobile elements (Spence et al., 2011).

Other peptidoglycan SNPs were previously reported to be associated with variations in niche adaption and virulence between closely related bacterial strains. The *rpoB* and *rpoC* genes code for the β- and β'-like subunits of the DNA-dependent RNA polymerase, which regulates gene expression in bacteria and archaea (Zakharova et al., 1998). Mutations in *rpoB* and *rpoC* genes were reported to be responsible for the different degrees of antibiotic resistance observed in *Staphylococcus aureus* (Matsuo et al., 2015), *Mycobacterium tuberculosis* (Ma et al., 2021) and *Clostridium difficile* (Kuehne et al., 2018). Similar observations on the impact of these mutations have been demonstrated for the virulence of *C. difficile* strains (Kuehne et al., 2018), as well as the differences in the ability of *E. coli* (Conrad et al., 2010) and *Helicobacter pylori* (Zakharova et al., 1998) to colonize different niches. SNPs in both *rpoB* and *cysS* were suggested to contribute to the niche adaptation of the multi-host pathogen *S. aureus* (Bacigalupe et al., 2019). The interaction between the “mutation frequency decline” (*mfd*) gene and RNA polymerase, for example, *rpoB*, plays a role in the development of antimicrobial resistance in highly divergent bacterial species (Ragheb et al., 2019). Additionally, *mfd* is recognized as the “pro-evolutionary factor,” as studies suggested that *mfd* plays a significant role in prokaryotic virulence and survival (Lindsey-Boltz and Sancar, 2021; Strick and Portman, 2019). Mutations on the outer membrane proteins, including *flgG* (Palau et al., 2021) and genes of the *hop* family (Linz et al., 2013), facilitated the niche adaptations of different strains *of Helicobacter pylori* and allowed the colonization of the new host. Non-synonymous mutations in the *mutS2*, a gene coding for endonuclease responsible for suppressing homologous recombination, were linked to increased mutation rates during niche adaptation in *Streptococcus pneumoniae* (Green et al., 2021) and species-specific variation among the *Aquilegia* species (Wang et al., 2022).

Each of the seven peptidoglycan SNPs consistently divided the 58 *C. fetus* strains into two distinct groups, CFF and CFV/CFVi, suggesting the potential of developing a reliable assay for the subspecies differentiation. The CFF or CFV/CFVi identification in this study is consistent with the reported identity of the *C. fetus* strains, except for P4531 (GCF_016406645.1). P4531 was announced as a *C. fetus* subsp. *venerealis* but was typed as a *C. fetus* subsp. *fetus* using the seven peptidoglycan SNPs in this study. Nonetheless, P4531 was previously identified as a *C. fetus* subsp. *venerealis* using a microbial identification system and 16S rRNA sequence analysis (Kim et al., 2021), which are not sufficient to accurately differentiate between the subspecies (van der Graaf-van Bloois et al., 2016a). In some cases where there was inconsistency between the reported phenotypic and genomic identification of the *C. fetus* subspecies, including cfvi03/596, 01/165, 99541, 98/v445, and 08/421, the identification using the seven peptidoglycan SNPs in this study demonstrated consistent alignment with the reported phenotypic identification, which was typed with the biochemical tests, glycine tolerance and H_2_S production, recommended by OIE.

Moreover, the identification of subspecies reported in this study was consistent with biochemical and other molecular tests. For example, the *C. fetus* subsp. *fetus* reference strain NCTC10842 (GCF_900475935.1) was reported as a CFF/CFVi (Farace et al., 2019) by the L-Cys transporter PCR assay. However, several phenotypic and genomic tests have confirmed the identity of NCTC10842 as a *C. fetus* subsp. *fetus*. A similar observation was observed at multiple strains, including NWU_ED23, 06/341, ADRI513, zaf3, 82-40, 04/554, H1-UY, and so on. A reliable and accurate identification of *C. fetus* subsp. *venerealis* biovars, which are commonly identified in the bovine genital tract, has been an objective goal in BGC diagnostics research. Hence, we propose the seven peptidoglycan SNPs described in this study could be potential tools for BGC diagnostics and differentiation from *C. fetus* subsp. *fetus*.

One limitation of this study was the number of *C. fetus* strains (*n* = 25) recruited for SNP calling. However, we carefully conducted the whole-genome comparison with only the complete genomes to avoid the potential of false positive results arising from incomplete genomes. Additionally, the peptidoglycan SNPs were further verified using all the annotated and contamination-free *C. fetus* genomes (*n* = 58) available on the NCBI RefSeq database, which is a curated database with a non-redundant, high-quality set of sequences with detailed annotations. Ideally, more *C. fetus* strains from multiple geographical regions and sources should be included in the whole-genome comparison to provide a more comprehensive view of the evolutionary events of *C. fetus* subspecies as well as the branching of *C. fetus* subsp. *venerealis* bv. intermedius at different geographical regions. To obtain a detailed evolutionary history of the genus *Campylobacter*, future investigations should also include the complete genomes of the other subspecies, such as *Campylobacter fetus* subsp. *testudinum*, as well as other closely related *Campylobacter* species. Additionally, the impacts of amino acid change on SNP-coding CDSs should be further investigated and validated with phenotypic assays to examine the effect on glycine tolerance.

## Conclusion

Our results have reinforced the high genetic stability of *C. fetus* subspecies, suggesting that they are at the early stage of their evolutionary history and their genomic diversity is at the nucleotide base level. Clonal evolution was found to have occurred separately within the non-Australian and Australian strains. Regardless of geographical regions, *C. fetus* subsp. *venerealis* and the biovar variants have potentially acquired common point mutations from the vertical transmission that have enabled their niche-specificity and pathogenicity that separates them from *C. fetus* subsp. *fetus*. The *C. fetus* subsp. *venerealis* and the biovar intermedius variant also acquired different SNPs. The peptidoglycan SNPs identified and verified in this study are key candidates for the development of more accurate multi-SNP genotyping assays because of their association with the differential glycine tolerance between *C. fetus* subsp. *fetus* and *C. fetus* subsp. *venerealis*.

## Data Availability

The datasets presented in this study can be found in online repositories. The names of the repository/repositories and accession number(s) can be found at: https://www.ncbi.nlm.nih.gov/, PRJNA675960.
