# Supplementary material for: Whole-genome comparison using complete genomes from Campylobacter fetus strains revealed single nucleotide polymorphisms on non-genomic islands for subspecies differentiation
Source: Front Microbiol. 2024 Sep 12;15:1452564. doi: 10.3389/fmicb.2024.1452564 (PMC11424552; doi:10.3389/fmicb.2024.1452564)
Supplement: Supplementary file 1 [file Data_Sheet_1.pdf]

Table S1: List of strains used for mraY PCR validation

Strain	Organism	Collection
ATCC 19438	*Campylobacter fetus* subsp. *venerealis* bv. venerealis	ATCC reference strain
A8	*Campylobacter fetus* subsp. *venerealis* bv. venerealis	Culture collection from the Queensland Alliance for Agriculture and Food Innovation at the University of Queensland
957	*Campylobacter fetus* subsp. *venerealis* bv. venerealis	Culture collection from the Queensland Alliance for Agriculture and Food Innovation at the University of Queensland
76223	*Campylobacter fetus* subsp. *venerealis* bv. intermedius	Culture collection from the Queensland Alliance for Agriculture and Food Innovation at the University of Queensland
924	*Campylobacter fetus* subsp. *venerealis* bv. intermedius	Culture collection from the Queensland Alliance for Agriculture and Food Innovation at the University of Queensland
926	*Campylobacter fetus* subsp. *venerealis* bv. intermedius	Culture collection from the Queensland Alliance for Agriculture and Food Innovation at the University of Queensland
ATCC 27374	*Campylobacter fetus* subsp. *fetus*	ATCC reference strain
BT376/03	*Campylobacter fetus* subsp. *fetus*	Culture collection from the Institute for Glycomics at the Griffith University
337	*Campylobacter hyointestinalis*	Culture collection from the Queensland Alliance for Agriculture and Food Innovation at the University of Queensland
312	*Arcobacter cryaerophilus*	Culture collection from the Queensland Alliance for Agriculture and Food Innovation at the University of Queensland
412	*Campylobacter ureolyticus*	Culture collection from the Queensland Alliance for Agriculture and Food Innovation at the University of Queensland
530	*Campylobacter sputorum*	Culture collection from the Queensland Alliance for Agriculture and Food Innovation at the University of Queensland

Table S2: Details of Oxford Nanopore Technologies sequencing output after quality filtering of *Campylobacter fetus* isolates sequenced in this study.

Sample ID	Species	Mean read length	Mean read quality	Median read length	Median read quality	Number of reads	Read length N50	Total bases
76223	*Campylobacter fetus* subsp. *venerealis*	25,847.20	12	18,759.50	12	10,198	31,389	263,589,743
924	*Campylobacter fetus* subsp. *venerealis* bv. intermedius	52,199.40	12.5	46,823.00	12.6	8,177	53,856	426,834,809

926	*Campylobacter fetus* subsp. *venerealis* bv. intermedius	26,063.20	11.8	19,630.00	11.8	14,823	31,605	386,335,515
957	*Campylobacter fetus* subsp. *venerealis*	49,858.60	13	44,632.50	13	18,062	50,929	900,546,509
A8	*Campylobacter fetus* subsp. *venerealis*	26,114.60	11.8	20,123.00	11.7	10,110	31,363	264,019,059
BT268/06	*Campylobacter fetus* subsp. *fetus*	30,181.20	13.6	25,865.00	13.6	15,682	38,806	473,301,472
BT376/3	*Campylobacter fetus* subsp. *fetus*	24,412.70	13.6	19,013.50	13.6	22,974	31,044	560,856,357
M20/08756-1A	*Campylobacter fetus* subsp. *fetus*	19,824.00	14.5	13,242	14.4	240,350	28,284	4,764,696,999
M20/04752-1B	*Campylobacter fetus* subsp. *fetus*	19,959.10	14	13,616	13.8	55,711	27,426	1,111,941,267

Table S3: List of Illumina shotgun sequencing output of *Campylobacter fetus* isolates sequenced in this study.

**ID**	**Species**	**Genome size (mb)**	**# of reads (Paired-end)**	**# of bases (Paired-end)**
A8	*Campylobacter fetus subsp. venerealis*	1.95	332,689	49,709,085
924	*Campylobacter fetus* subsp. *venerealis* bv. intermedius	1.95	419,514	62,681,945
926	*Campylobacter fetus* subsp. *venerealis* bv. intermedius	1.95	473,809	70,993,988
957	*Campylobacter fetus subsp. venerealis*	1.95	469,778	70,267,478
76223	*Campylobacter fetus subsp. venerealis*	1.95	388,562	58,188,902
M20/08756-1A	*Campylobacter fetus* subsp. *fetus*	1.79	581,146	87,171,900
M20/04752-1B	*Campylobacter fetus* subsp. *fetus*	1.79	552,446	82,866,900

Table S4: QUAST report on the assembly qualities of each *Campylobacter fetus* isolates assessed using the long reads.

	**ATCC 19438**	**ATCC 27374**	**BT268/06**	**BT376/03**	**924**	**926**	**957**	**76223**	**A8**	**M20/08756-1A**	**M20/04752-1B**
# reads	104861	116743	14078	10418	13651	20517	22686	18139	13170	672198	258159
# contigs	1	1	1	1	1	1	1	1	1	1	1
Largest contig	1833442	1763261	1909714	1800589	2250778	2123600	2088026	2105546	2112436	1782221	1782237
Total length	1833442	1763261	1909714	1800589	2250778	2123600	2088026	2105546	2112436	1782221	1782237
N50	1833442	1763261	1909714	1800589	2250778	2123600	2088026	2105546	2112436	1782221	1782237
N75	1833442	1763261	1909714	1800589	2250778	2123600	2088026	2105546	2112436	1782221	1782237
L50	1	1	1	1	1	1	1	1	1	1	1
L75	1	1	1	1	1	1	1	1	1	1	1
GC (%)	33.25	33.29	33.26	33.22	33.48	33.48	33.41	33.47	33.42	33.1	33.31
**Reads mapping**											
# mapped	104543	116512	14078	10418	13651	20467	22686	18123	13131	671858	221025
Mapped (%)	99.7	99.8	100	100	100	99.76	100	99.91	99.7	99.95	85.62
# properly paired	0	0	0	0	0	0	0	0	0	0	0
Properly paired (%)	0	0	0	0	0	0	0	0	0	0	0
# singletons	0	0	0	0	0	0	0	0	0	0	0
Singletons (%)	0	0	0	0	0	0	0	0	0	0	0
# misjoint mates	0	0	0	0	0	0	0	0	0	0	0
Misjoint mates (%)	0	0	0	0	0	0	0	0	0	0	0
Avg. coverage depth	408	484	344	231	252	206	451	186	135	4257	1249
Coverage >= 1x (%)	100	100	100	100	100	100	100	100	100	100	100
Coverage >= 5x (%)	100	100	100	100	100	100	100	100	100	100	100
Coverage >= 10x (%)	100	100	100	100	100	100	100	100	100	100	100

Table S5: QUAST report on the assembly qualities of each *Campylobacter fetus* isolates assessed using the short reads.

**Statistics without reference**	**ATCC 19438**	**ATCC 27374**	**924**	**926**	**957**	**76223**	**A8**	**M20/08756-1A**	**M20/04752-1B**
# reads	840,503	620,165	839,445	947,831	939,771	777,335	665,611	1,162,923	1,106,439
# contigs	1	1	1	1	1	1	1	1	1
Largest contig	1,833,442	1,763,261	2,250,778	2,123,600	2,088,026	2,105,546	2,112,436	1,782,221	1,782,237
Total length	1,833,442	1,763,261	2,250,778	2,123,600	2,088,026	2,105,546	2,112,436	1,782,221	1,782,237
N50	1,833,442	1,763,261	2,250,778	2,123,600	2,088,026	2,105,546	2,112,436	1,782,221	1,782,237
N75	1,833,442	1,763,261	2,250,778	2,123,600	2,088,026	2,105,546	2,112,436	1,782,221	1,782,237
L50	1	1	1	1	1	1	1	1	1
L75	1	1	1	1	1	1	1	1	1
GC (%)	33.25	33.29	33.48	33.48	33.41	33.47	33.42	33.31	33.1
**Reads mapping**									
# mapped	802,439	619,156	798,266	918,744	911,289	748,716	645,559	1,162,123	1,105,643
Mapped (%)	95.47	99.84	95.09	96.93	96.97	96.32	96.99	99.93	99.93
# properly paired	798,158	616,250	793,172	914,148	907,020	745,856	642,246	1,158,380	1,100,508
Properly paired (%)	94.96	99.37	94.49	96.45	96.52	95.95	96.49	99.61	99.46
# singletons	2000	651	1657	1409	1632	1153	1092	712	720
Singletons (%)	0.24	0.1	0.2	0.15	0.17	0.15	0.16	0.06	0.07
# misjoint mates	0	0	0	0	0	0	0	0	0
Misjoint mates (%)	0	0	0	0	0	0	0	0	0
Avg. coverage depth	65	52	52	64	65	53	45	95	90
Coverage >= 1x (%)	100	100	98.3	98.58	99.38	99.21	98.94	100	100
Coverage >= 5x (%)	100	100	96.65	97.65	98.75	98.26	97.7	100	99.99
Coverage >= 10x (%)	100	100	95.19	96.92	98.14	97.33	96.64	100	99.99

Table S6: Results reported by annotating the 25 *Campylobacter fetus* isolates using Prokka.

ID	Accession	Organism	Gene	rRNA	tRNA	tmRNA	CDS	Repeat region	Contigs
BT268/06		*Campylobacter fetus* subsp. *fetus*	2092	6	44	1	2041	1	1
BT376/03		*Campylobacter fetus* subsp. *fetus*	2105	6	44	1	2054	1	1
924		*Campylobacter fetus* subsp. *venerealis* bv. intermedius	2372	6	44	1	2321	1	2
926		*Campylobacter fetus* subsp. *venerealis* bv. intermedius	2330	6	44	1	2279	1	2
957		*Campylobacter fetus* subsp. *venerealis*	2208	6	44	1	2157	1	2
76223		*Campylobacter fetus* subsp. *venerealis* bv. intermedius	2249	6	44	1	2198	1	2
A8		*Campylobacter fetus* subsp. *venerealis*	2324	6	44	1	2273	1	2
NCTC10842	GCA_900475935.1	*Campylobacter fetus* subsp. *fetus*	1785	6	44	1	1734	2	1
NCTC10354	GCA_008271385.1	*Campylobacter fetus* subsp. *venerealis*	1985	6	44	1	1934	1	2
82-40	GCA_000015085.1	*Campylobacter fetus* subsp. *fetus*	1789	6	44	1	1738	2	1
84-112	GCA_000967135.1	*Campylobacter fetus* subsp. *venerealis*	2076	6	44	1	2025	1	2
ADRI545	GCA_011601375.2	*Campylobacter fetus* subsp. *venerealis* bv. intermedius	2216	6	44	1	2165	1	2
97-608	GCA_000759515.1	*Campylobacter fetus* subsp. *fetus*	2094	6	44	1	2043	1	3
ADRI1362	GCA_011600955.2	*Campylobacter fetus* subsp. *venerealis* bv. intermedius	2369	6	44	1	2318	1	5
00A031	GCA_011600945.2	*Campylobacter fetus* subsp. *fetus*	1826	6	44	1	1775	2	2
01/165	GCA_001686885.1	*Campylobacter fetus* subsp. *venerealis*	1999	6	43	1	1949	1	3
02A725-35A	GCA_011600855.2	*Campylobacter fetus* subsp. *fetus*	1866	6	44	1	1815	1	1
03-293	GCA_000512745.2	*Campylobacter fetus* subsp. *venerealis* bv. intermedius	2078	6	44	1	2027	1	4
04-554	GCA_000759485.1	*Campylobacter fetus* subsp. *fetus*	1904	6	44	1	1853	1	2
08A948-2A	GCA_011601005.2	*Campylobacter fetus* subsp. *venerealis*	2088	6	44	1	2037	1	2
08A1102-42A	GCA_011600845.2	*Campylobacter fetus* subsp. *venerealis*	2088	6	44	1	2037	1	2
09A980	GCA_011600995.2	*Campylobacter fetus* subsp. *fetus*	1898	6	44	1	1847	2	2
INIA/17144	GCA_007723545.1	*Campylobacter fetus* subsp. *fetus*	1902	6	44	1	1851	2	1
M20/08756-1A		*Campylobacter fetus* subsp. *fetus*	1875	6	44	1	1824	0	1
M20/04752-1B		*Campylobacter fetus* subsp. *fetus*	1869	6	44	1	1818	0	1

Table S7. Presence or absence of gene families encoding for the peptidase S24 LexA-like protein among the 25 *C. fetus* strains. International Organization for Standardization (ISO) country code: Canada (CA), United States (US), France (FR), Uruguay (UY), Argentina (AR), United Kingdom (UK), Australia (AU) and New Zealand (NZ).

**Strain**	**Gene family**	**group_129***	**group_17***	**group_680***	**group_650***
	**Annotation**	**peptidase S24 LexA-like protein**	**peptidase S24 LexA-like protein**	**peptidase S24 LexA-like protein**	**peptidase S24 LexA-like protein**
C. fetus subsp. *fetus*	BT268/06 (UK)	No	Yes	No	No
C. fetus subsp. *fetus*	BT376/03 (UK)	No	Yes	No	No
C. fetus subsp. *fetus*	GCA_000015085 (US)	No	No	No	No
C. fetus subsp. *fetus*	GCA_000759485 (AR)	No	Yes	No	No
C. fetus subsp. *fetus*	GCA_007723545 (UY)	No	Yes	No	No
C. fetus subsp. *fetus*	GCA_011600855 (CA)	No	Yes	No	No
C. fetus subsp. *fetus*	GCA_011600945 (CA)	No	No	No	No
C. fetus subsp. *fetus*	GCA_011600995 (CA)	No	No	No	No
C. fetus subsp. *fetus*	GCA_900475935^T (FR)	No	No	No	No
C. fetus subsp. *fetus*	M20-04752-1B (AU)	No	Yes	No	No
C. fetus subsp. *fetus*	M20-08756-1A (NZ)	No	Yes	No	No
C. fetus subsp. *venerealis*	76223 (AU)	Yes	Yes	Yes	Yes
C. fetus subsp. *venerealis*	957 (AU)	Yes	Yes	Yes	Yes
C. fetus subsp. *venerealis*	A8 (AU)	Yes	Yes	Yes	Yes
C. fetus subsp. *venerealis*	GCA_000759515 (AR)	Yes	Yes	No	Yes
C. fetus subsp. *venerealis*	GCA_000967135 (US)	Yes	Yes	No	Yes
C. fetus subsp. *venerealis*	GCA_008271385^T (UK)	Yes	Yes	No	Yes
C. fetus subsp. *venerealis*	GCA_011600845 (CA)	Yes	Yes	No	Yes
C. fetus subsp. *venerealis*	GCA_011601005 (CA)	Yes	Yes	No	Yes
C. fetus subsp. *venerealis* bv. intermedius	924 (AU)	Yes	Yes	Yes	No
C. fetus subsp. *venerealis* bv. intermedius	926 (AU)	Yes	Yes	Yes	Yes
C. fetus subsp. *venerealis* bv. intermedius	GCA_000512745 (AR)	Yes	No	No	No
C. fetus subsp. *venerealis* bv. intermedius	GCA_001686885 (AR)	Yes	No	No	No
C. fetus subsp. *venerealis* bv. intermedius	GCA_011600955 (AR)	Yes	Yes	No	Yes
C. fetus subsp. *venerealis* bv. intermedius	GCA_011601375 (AU)	Yes	Yes	Yes	Yes

*The ortholog group name were assigned by Roary and is different in each run.

Table S8: List of candidate single nucleotide polymorphisms (SNPs) in the 25 *Campylobacter fetus* isolates which were differentiative between *C. fetus fetus* and *C. fetus venerealis*, causing non-synonymous amino acid change and outside of both recombination region and putative genomics islands.

**SNP position**	**Gene/Locus tag**	**Product**	**REF**	**ALT**
9551	flgE	flagellar hook protein	A	C
24341	nadV	nicotinamide phosphoribosyltransferase	T	C
72154	fliD	flagellar filament cap protein	G	T
109019	hyfB	hydrogenase-4, transmembrane adaptor	T	G
110191	hyfB	hydrogenase-4, transmembrane adaptor	T	C
111648	non-coding	between hyfA and CFVT_0136	A	G
118976	CFVT_0140	NapC/NirT cytochrome c family protein	G	T
124064	CFVT_0148	anaerobic C4-dicarboxylate transporter,DcuA/DcuB family	A	C
129731	CFVT_0153	hypothetical protein	G	A
159398	CFVT_0187	MCP-domain signal transduction protein	C	T
160779	moeA1	molybdopterin molybdenumtransferase	A	C
161602	non-coding	between rimI and CFVT_0191	C	T
161989	CFVT_0191	putative selenium metabolism protein, YedEfamily	A	G
183993	pflA	medial disk protein PflA	A	G
207138	gppA	guanosine-5'-triphosphate, 3'-diphosphate pyrophosphatase	A	G
215285	xseA	exodeoxyribonuclease VII, large subunit	A	G
218548	CFVT_0259	putative dicarboxylate carrier protein MatC	A	G
227587	fliG	flagellar motor switch C-ring protein FliG	A	T
230497	hisC	histidinol-phosphate aminotransferase	C	T
233133	lysA	diaminopimelate decarboxylase	A	G
235240	non-coding	between iptG and CFVT_0274	A	G
237990	CFVT_0276	TonB-dependent receptor	A	G
266258	CFVT_0312	DUF748 domain-containing protein	C	T
299951	uvrA	UvrABC nucleotide excision repair complex,subunit UvrA	A	T
303623	CFVT_0348	DUF302 domain-containing protein	C	T
311221	CFVT_0356	putative membrane protein	A	G
318313	CFVT_0365	RND superfamily transporter	T	C
318852	CFVT_0365	RND superfamily transporter	A	G
328158	CFVT_0373	DUF507 domain-containing protein	T	C
350208	CFVT_0394	hypothetical protein	G	A
350229	CFVT_0394	hypothetical protein	G	A
351067	non-coding	between CFVT_0395 and cmp	T	C
351177	non-coding	between CFVT_0395 and cmp	A	T
358672	ccsBA	cytochrome c synthetase	A	C
369503	CFVT_0412	ABC transporter, ATP-binding protein	A	G

SNP position	Gene/Locus tag	Product	REF	ALT
376029	dinP	DNA polymerase IV	T	C
381600	CFVT_0423	YebC/PmpR family DNA-binding regulatory protein	C	T
391070	CFVT_0433	TonB-dependent receptor	A	G
453864	flgG	flagellar distal rod protein FlgG	G	A
459299	CFVT_0558	ferrirhodotorulic acid ABC transporter, permeaseprotein	T	C
460434	CFVT_0559	ferrirhodotorulic acid ABC transporter, permeaseprotein	T	C
461013	CFVT_0560	ferrirhodotorulic acid ABC transporter,ATP-binding protein	T	C
468294	CFVT_0568	two-component system sensor histidine kinase	T	G
473580	CFVT_0572	sodium-dependent transporter, SNF family	C	T
481044	amt	ammonium transporter	T	C
489435	djlA	DnaJ-like membrane chaperone protein	G	A
519887	glnD	protein-P-II uridylyltransferase	G	A
522955	fdhT	formate dehydrogenase biogenesis protein FdhT	A	G
542808	murC	UDP-N-acetylmuramate-alanine ligase	A	G
550629	mutS2	DNA mismatch binding protein, MutS2 family	T	C
555222	mqo	malate:quinone-oxidoreductase	T	C
555759	mqo	malate:quinone-oxidoreductase	A	G
556216	CFVT_0660	SAM-dependent methyltransferase	A	G
566302	CFVT_0667	hypothetical protein	T	C
570811	CFVT_0672	histidine triad nucleotide-binding protein, Fhitbranch	A	G
600426	groEL	60 kD chaperonin (cpn60)	G	A
601760	hemH	ferrochelatase	G	A
602421	ftsI	cell division protein FtsI / penicillin-bindingprotein	G	T
623077	CFVT_0728	metal ion ABC transporter, permease protein	G	A
635901	mfd	transcription-repair coupling factor	T	C
638033	CFVT_0741	SprA family protein	T	C
642926	CFVT_0747	hemagglutinin domain-containing protein	C	T
666793	non-coding	between cprR and htrA	A	G
670791	CFVT_0769	potassium:proton antiporter; gene disrupted byISCfvt2A	G	T
671945	CFVT_0772	potassium:proton antiporter	A	G
677769	gpsA	glycerol-3-phosphate dehydrogenase	A	G
688237	cysS	cysteinyl-tRNA synthetase	T	C
709030	CFVT_0811	alpha/beta hydrolase family protein	A	G
713204	pta	phosphate acetyltransferase	T	C
714699	CFVT_0816	DUF305 domain-containing protein	T	C
722598	CFVT_0825	endonuclease/exonuclease/phosphatase	T	C
745673	CFVT_0861	RseP-like zinc metalloprotease, M50 family	T	G
746365	non-coding	between CFVT_0861 and rpe	A	G
750272	CFVT_0866	putative type II secretion system protein	C	T
750581	CFVT_0866	putative type II secretion system protein	A	C

SNP position	Gene/Locus tag	Product	REF	ALT
750584	CFVT_0866	putative type II secretion system protein	G	T
750601	CFVT_0866	putative type II secretion system protein	G	A
750656	CFVT_0866	putative type II secretion system protein	A	G
762235	non-coding	between ppk and CFVT_0876	G	A
809995	CFVT_0925	AsmA family protein (DUF3971 domain)	T	C
814691	hypB	hydrogenase nickel insertion protein HypB	T	C
855307	CFVT_0971	short-chain dehydrogenase/reductase, subgroup 5	C	T
864687	CFVT_0986	DUF3519 domain-containing protein; gene disruptedby ISCfvt2B	C	T
864721	CFVT_0986	DUF3519 domain-containing protein; gene disruptedby ISCfvt2B	G	A
865378	ctb	group III truncated hemoglobin	T	C
868219	acnB	aconitate hydratase 2	C	T
871555	ribB	3,4-dihydroxy-2-butanone-4-phosphate synthase	A	C
874971	era	GTP-binding protein	A	G
877290	CFVT_1000	hypothetical protein	A	C
878437	ctsD	transformation system protein CtsD	T	C
885295	CFVT_1007	MCP-domain signal transduction protein	T	C
886089	CFVT_1009	exopolyphosphatase, Ppx/GppA family	A	C
890677	CFVT_1016	transporter, LysE family	C	T
892158	ansB	asparaginase II	C	T
894336	non-coding	between rmuC and CFVT_1020	C	A
919675	CFVT_1047	hypothetical protein	T	C
924449	non-coding	between CFVT_1050 and lon	G	A
924848	lon	DNA-binding, ATP-dependent protease La	G	A
932160	fucP	L-fucose permease	T	G
938813	cmoB	tRNA (cmo5U34)-carboxymethyltransferase	G	T
946534	CFVT_1074	putative nitrous oxide-regulated protein	A	G
949387	dsbD	thiol:disulfide interchange protein DsbD	A	G
953934	grpE	DnaK system nucleotide exchange factor GrpE	A	C
964164	potC	iron(III)/spermidine/putrescine ABC transporter,permease protein	T	C
969998	ligA	DNA ligase, NAD-dependent	G	A
991570	racS	two-component system sensor histidine kinase	A	G
1017123	ychF	GTP-binding protein, putative GTP-dependenttranslation factor	T	C
1025349	CFVT_1167	putative membrane protein	G	T
1028052	CFVT_1169	hypothetical protein	T	G
1069376	CFVT_1212	MCP-domain signal transduction protein	A	G
1075287	CFVT_1271	sodium:proline symporter	T	C
1079686	thiE	thiamine phosphate synthase (TMP-TENI domain)	G	A
1080605	bacA	undecaprenyl pyrophosphate phosphatase	C	T
1089084	putA	proline dehydrogenase /1-pyrroline-5-carboxylate dehydrogenase	G	A
1112095	lptD	lipooligosaccharide transport system, OMtranslocon component LptD	T	C

SNP position	Gene/Locus tag	Product	REF	ALT
1133066	msrAB	bifunctional (RS)-methionine sulfoxide reductaseA/B	C	T
1138970	CFVT_1329	iron-containing alcohol dehydrogenase	G	A
1141321	non-coding	between CFVT_1332 and CFVT_1333	G	A
1153488	non-coding	between CFVT_1342 and cueO	A	G
1161230	rpoC	DNA-directed RNA polymerase, beta' subunit	C	T
1164748	rpoC	DNA-directed RNA polymerase, beta' subunit	A	G
1165317	rpoB	DNA-directed RNA polymerase, beta subunit	T	C
1166903	rpoB	DNA-directed RNA polymerase, beta subunit	T	C
1172704	tuf	translation elongation factor Tu	C	T
1180110	engA	GTP-binding protein	T	C
1195278	pglF	UDP-N-acetylglucosamine C-6 dehydratase	C	T
1199164	mraY	phospho-N-acetylmuramoyl-pentapeptidetransferase	T	C
1204296	accA	acetyl-CoA carboxylase, carboxyltransferase,alpha subunit	G	A
1209583	CFVT_1403	radical SAM methylthiotransferase, MiaB/RimOfamily	T	C
1211960	CFVT_1406	hypothetical protein	G	T
1214232	miaB	isopentenyl-adenosine A37 tRNA methylthiolase	T	G
1214311	miaB	isopentenyl-adenosine A37 tRNA methylthiolase	A	C
1214719	miaB	isopentenyl-adenosine A37 tRNA methylthiolase	T	C
1216175	nusA	transcription termination factor	T	C
1216343	nusA	transcription termination factor	A	C
1223454	CFVT_1418	glycosyltransferase, family 1	A	G
1230523	CFVT_1429	polysaccharide biosynthesis protein, nucleotidesugar dehydrogenase, TviB family	A	G
1233431	waaC	heptosyltransferase I	A	C
1237772	CFVT_1436	glycosyltransferase, family 9	A	G
1238014	CFVT_1436	glycosyltransferase, family 9	A	G
1263785	pgk	phosphoglycerate kinase	A	G
1287937	hemE	uroporphyrinogen decarboxylase	A	G
1290625	flgR	flagella-associated two-component system,response regulator	C	T
1293352	gyrA	DNA gyrase, subunit A	C	T
1295506	CFVT_1537	aminoglycoside N3'-acetyltransferase	A	G
1295937	CFVT_1537	aminoglycoside N3'-acetyltransferase	T	C
1297236	CFVT_1540	AMP-forming adenylation domain superfamilyprotein, putative D-alanine:D-alanyl carrier protein ligase	C	T
1298139	CFVT_1540	AMP-forming adenylation domain superfamilyprotein, putative D-alanine:D-alanyl carrier protein ligase	A	G
1302569	lepA	elongation factor 4	G	A
1305827	pyrB	aspartate carbamoyltransferase, catalyticsubunit	G	A
1308981	mnmA	tRNA U34 2-thiouridylase	T	C
1309323	non-coding	between mnmA and fdhC	C	T
1309372	non-coding	between mnmA and fdhC	A	G
1311183	CFVT_1555	putative lysine decarboxylase family protein	C	T

SNP position	Gene/Locus tag	Product	REF	ALT
1311656	non-coding	between CFVT_1555 and gltD	C	T
1311843	gltD	glutamate synthase, small subunit	G	A
1324951	CFVT_1568	epimerase, PhzC/PhzF family	T	C
1340355	fliY	flagellar motor switch C-ring protein FliY	A	G
1377030	gdhA	glutamate dehydrogenase	G	A
1379674	mez	malate oxidoreductase	C	T
1380133	mez	malate oxidoreductase	A	C
1381712	gltX2	glutamyl-tRNA synthetase	T	G
1381736	gltX2	glutamyl-tRNA synthetase	G	A
1395656	CFVT_1646	hypothetical protein	T	G
1398196	CFVT_1650	hypothetical protein	T	C
1413687	CFVT_1673	asparagine synthase (glutamine-hydrolyzing)	C	T
1413889	CFVT_1675	hypothetical protein	T	C
1417766	CFVT_1679	asparagine synthase (glutamine-hydrolyzing)	A	G
1418311	CFVT_1679	asparagine synthase (glutamine-hydrolyzing)	G	A
1419087	CFVT_1680	DUF354 domain-containing protein	T	C
1447152	CFVT_1710	ferric enterobactin uptake receptor CfrA	A	C
1448019	non-coding	between CFVT_1710 and exbB3	A	G
1448939	tonB3	energy transduction protein TonB	A	G
1491313	CFVT_1762	hemerythrin	T	C
1491460	CFVT_1762	hemerythrin	A	G
1502101	CFVT_1777	TM2 domain-containing protein	C	T
1503995	glmM	phosphoglucosamine mutase	G	T
1506068	CFVT_1783	YcaC-related amidohydrolase	T	C
1506817	xth	exodeoxyribonuclease III (exonuclease III)	T	C
1536899	ushA	bifunctional UDP-sugar hydrolase /5'-nucleotidase periplasmic precursor	A	G
1536975	ushA	bifunctional UDP-sugar hydrolase /5'-nucleotidase periplasmic precursor	T	C
1556937	non-coding	between coaE and CFVT_1867	T	C
1567780	CFVT_1884	autotransporter serine protease (peptidase_S8domain)	A	C
1577565	CFVT_1895	DoxX family protein	A	G

Table S9: List of COG functional annotations of the candidate SNPs identified from the 25 *Campylobacter fetus* isolates.

**Query**	**COG category**	**COG functional classification**	**COG general categories**
1017123_1156667_ychF_GTP-binding	J	Translation, ribosomal structure and biogenesis	Information storage and processing
1025349_1164897_CFVT_1167_putative	-		Unclassified
1028052_1167605_CFVT_1169_hypothetical	-		Unclassified
1069376_1208996_CFVT_1212_MCP-domain	NT		Cellular processes and signaling
1075287_1256704_CFVT_1271_sodium:proline	E	Amino acid transport and metabolism	Metabolism
1079686_1261096_thiE_thiamine	H	Coenzyme transport and metabolism	Metabolism
1080605_1262015_bacA_undecaprenyl	V	Defense mechanisms	Cellular processes and signaling
1089084_1270509_putA_proline	C	Energy production and conversion	Metabolism
109019_111972_110191_113144_hyfB_hydrogenase-4,	CP		Metabolism
1112095_1293549_lptD_lipooligosaccharide	M	Cell wall/membrane/envelope biogenesis	Cellular processes and signaling
1133066_1314525_msrAB_bifunctional	O	Post-translational modification, protein turnover, and chaperones	Cellular processes and signaling
1138970_1320429_CFVT_1329_iron-containing	C	Energy production and conversion	Metabolism
1161230_1345830_1164748_1349348_rpoC_DNA-directed	K	Transcription	Information storage and processing
1165317_1349917_1166903_1351503_rpoB_DNA-directed	K	Transcription	Information storage and processing
1172704_1357304_tuf_translation	J	Translation, ribosomal structure and biogenesis	Information storage and processing
1180110_1365273_engA_GTP-binding	S	Function unknown	Poorly characterized
118976_121957_CFVT_0140_NapC/NirT	C	Energy production and conversion	Metabolism
1195278_1380446_pglF_UDP-N-acetylglucosamine	GM		Multiple
1199164_1384375_mraY_phospho-N-acetylmuramoyl-pentapeptidetransferase	M	Cell wall/membrane/envelope biogenesis	Cellular processes and signaling
1204296_1389507_accA_acetyl-CoA	I	Lipid transport and metabolism	Metabolism
1209583_1395256_CFVT_1403_radical	J	Translation, ribosomal structure and biogenesis	Information storage and processing
1211960_1397635_CFVT_1406_hypothetical	NU		Cellular processes and signaling
1214232_1399907_1214311_1399986_1214719_1400394_miaB_isopentenyl-adenosine	J	Translation, ribosomal structure and biogenesis	Information storage and processing
1216175_1401850_1216343_1402018_nusA_transcription	K	Transcription	Information storage and processing

Query	COG category	COG functional classification	COG general categories
1223454_1409129_CFVT_1418_glycosyltransferase,	M	Cell wall/membrane/envelope biogenesis	Cellular processes and signaling
1230523_1423166_CFVT_1429_polysaccharide	M	Cell wall/membrane/envelope biogenesis	Cellular processes and signaling
1233431_1426074_waaC_heptosyltransferase	M	Cell wall/membrane/envelope biogenesis	Cellular processes and signaling
1237772_1430415_1238014_1430657_CFVT_1436_glycosyltransferase,	M	Cell wall/membrane/envelope biogenesis	Cellular processes and signaling
124064_127047_CFVT_0148_anaerobic	P	Inorganic ion transport and metabolism	Metabolism
1263785_1457064_pgk_phosphoglycerate	F	Nucleotide transport and metabolism	Metabolism
1287937_1512653_hemE_uroporphyrinogen	H	Coenzyme transport and metabolism	Metabolism
1290625_1515342_flgR_flagella-associated	T	Signal transduction mechanisms	Cellular processes and signaling
1293352_1518069_gyrA_DNA	L	Replication, recombination and repair	Information storage and processing
1295506_1520226_1295937_1520657_CFVT_1537_aminoglycoside	V	Defense mechanisms	Cellular processes and signaling
1297236_1521956_1298139_1522859_CFVT_1540_AMP-forming	Q	Secondary metabolites biosynthesis, transport, and catabolism	Metabolism
129731_132719_CFVT_0153_hypothetical	-		Unclassified
1302569_1527295_lepA_elongation	M	Cell wall/membrane/envelope biogenesis	Cellular processes and signaling
1305827_1530556_pyrB_aspartate	F	Nucleotide transport and metabolism	Metabolism
1308981_1534081_mnmA_tRNA	J	Translation, ribosomal structure and biogenesis	Information storage and processing
1311183_1536283_CFVT_1555_putative	S	Function unknown	Poorly characterized
1311843_1536943_gltD_glutamate	C	Energy production and conversion	Metabolism
1324951_1550298_CFVT_1568_epimerase,	S	Function unknown	Poorly characterized
1340355_1565717_fliY_flagellar	N	Cell motility	Cellular processes and signaling
1377030_1604705_gdhA_glutamate	E	Amino acid transport and metabolism	Metabolism
1379674_1607392_1380133_1607831_mez_malate	C	Energy production and conversion	Metabolism
1381712_1609410_1381736_1609434_gltX2_glutamyl-tRNA	J	Translation, ribosomal structure and biogenesis	Information storage and processing
1395656_1623474_CFVT_1646_hypothetical protein	-	-	Unclassified
1398196_1626014_CFVT_1650_hypothetical	-		Unclassified
1413687_1650694_CFVT_1673_asparagine	E	Amino acid transport and metabolism	Metabolism

Query	COG category	COG functional classification	COG general categories
1413889_1650896_CFVT_1675_hypothetical protein	-	-	Unclassified
1417766_1656961_1418311_1657506_CFVT_1679_asparagine	E	Amino acid transport and metabolism	Metabolism
1419087_1658282_CFVT_1680_DUF354	S	Function unknown	Poorly characterized
1447152_1695933_CFVT_1710_ferric	P	Inorganic ion transport and metabolism	Metabolism
1448939_1697803_tonB3_energy	U	Intracellular trafficking, secretion, and vesicular transport	Cellular processes and signaling
1491313_1742515_1491460_1742662_CFVT_1762_hemerythrin_bacteriohemerythrin	P	Inorganic ion transport and metabolism	Metabolism
1502101_1754227_CFVT_1777_TM2	S	Function unknown	Poorly characterized
1503995_1756122_glmM_phosphoglucosamine	G	Carbohydrate transport and metabolism	Metabolism
1506068_1758195_CFVT_1783_YcaC-related	Q	Secondary metabolites biosynthesis, transport, and catabolism	Metabolism
1506817_1758944_xth_exodeoxyribonuclease	L	Replication, recombination and repair	Information storage and processing
1536899_1789816_1536975_1789892_ushA_bifunctional	F	Nucleotide transport and metabolism	Metabolism
1567780_1855468_CFVT_1884_autotransporter	O	Post-translational modification, protein turnover, and chaperones	Cellular processes and signaling
1577565_1865260_CFVT_1895_DoxX	S	Function unknown	Poorly characterized
159398_168740_CFVT_0187_MCP-domain	NT		Cellular processes and signaling
160779_171190_moeA1_molybdopterin	H	Coenzyme transport and metabolism	Metabolism
161989_173177_CFVT_0191_putative	S	Function unknown	Poorly characterized
183993_196802_pflA_medial	N	Cell motility	Cellular processes and signaling
207138_220497_gppA_guanosine-5_-triphosphate,	FP		Metabolism
215285_228650_xseA_exodeoxyribonuclease	L	Replication, recombination and repair	Information storage and processing
218548_231913_CFVT_0259_putative	P	Inorganic ion transport and metabolism	Metabolism
227587_241344_fliG_flagellar	N	Cell motility	Cellular processes and signaling
230497_244254_hisC_histidinol-phosphate	E	Amino acid transport and metabolism	Metabolism
233133_246890_lysA_diaminopimelate	E	Amino acid transport and metabolism	Metabolism
237990_251748_CFVT_0276_TonB-dependent	P	Inorganic ion transport and metabolism	Metabolism
24341_24767_nadV_nicotinamide	H	Coenzyme transport and metabolism	Metabolism
266258_280674_CFVT_0312_DUF748	S	Function unknown	Poorly characterized
299951_315447_uvrA_UvrABC	L	Replication, recombination and repair	Information storage and processing

Query	COG category	COG functional classification	COG general categories
303623_319119_CFVT_0348_DUF302	S	Function unknown	Poorly characterized
311221_326720_CFVT_0356_putative	N	Cell motility	Cellular processes and signaling
318313_333816_318852_334355_CFVT_0365_RND	S	Function unknown	Poorly characterized
328158_343661_CFVT_0373_DUF507	S	Function unknown	Poorly characterized
350208_365731_350229_365752_CFVT_0394_hypothetical	-		Unclassified
358672_374621_ccsBA_cytochrome	O	Post-translational modification, protein turnover, and chaperones	Cellular processes and signaling
369503_385452_CFVT_0412_ABC	S	Function unknown	Poorly characterized
376029_391976_dinP_DNA	L	Replication, recombination and repair	Information storage and processing
381600_397547_CFVT_0423_YebC/PmpR	K	Transcription	Information storage and processing
391070_408725_CFVT_0433_TonB-dependent	P	Inorganic ion transport and metabolism	Metabolism
453864_549585_flgG_flagellar	N	Cell motility	Cellular processes and signaling
459299_555047_CFVT_0558_ferrirhodotorulic	V	Defense mechanisms	Cellular processes and signaling
460434_556182_CFVT_0559_ferrirhodotorulic	V	Defense mechanisms	Cellular processes and signaling
461013_556761_CFVT_0560_ferrirhodotorulic	V	Defense mechanisms	Cellular processes and signaling
468294_564042_CFVT_0568_two-component	T	Signal transduction mechanisms	Cellular processes and signaling
473580_569331_CFVT_0572_sodium-dependent	P	Inorganic ion transport and metabolism	Metabolism
481044_576795_amt_ammonium	P	Inorganic ion transport and metabolism	Metabolism
489435_585186_djlA_DnaJ-like	O	Post-translational modification, protein turnover, and chaperones	Cellular processes and signaling
519887_616192_glnD_protein-P-II	O	Post-translational modification, protein turnover, and chaperones	Cellular processes and signaling
522955_619264_fdhT_formate	S	Function unknown	Poorly characterized
542808_646169_murC_UDP-N-acetylmuramate-alanine	M	Cell wall/membrane/envelope biogenesis	Cellular processes and signaling
550629_653990_mutS2_DNA	L	Replication, recombination and repair	Information storage and processing
555222_658583_555759_659120_mqo_malate:quinone-oxidoreductase	C	Energy production and conversion	Metabolism
556216_659577_CFVT_0660_SAM-dependent	S	Function unknown	Poorly characterized
566302_669756_CFVT_0667_hypothetical	D	Cell cycle control, cell division, chromosome partitioning	Cellular processes and signaling
570811_674265_CFVT_0672_histidine	FG		Metabolism

**Query**	**COG category**	**COG functional classification**	**COG general categories**
600426_707841_groEL_60	O	Post-translational modification, protein turnover, and chaperones	Cellular processes and signaling
601760_709618_hemH_ferrochelatase	H	Coenzyme transport and metabolism	Metabolism
602421_710279_ftsI_cell	M	Cell wall/membrane/envelope biogenesis	Cellular processes and signaling
623077_731192_CFVT_0728_metal	U	Intracellular trafficking, secretion, and vesicular transport	Cellular processes and signaling
635901_744016_mfd_transcription-repair	L	Replication, recombination and repair	Information storage and processing
638033_746148_CFVT_0741_SprA	M	Cell wall/membrane/envelope biogenesis	Cellular processes and signaling
642926_751461_CFVT_0747_hemagglutinin	U	Intracellular trafficking, secretion, and vesicular transport	Cellular processes and signaling
670791_782001_CFVT_0769_potassium:proton	L	Replication, recombination and repair	Information storage and processing
671945_783155_CFVT_0772_potassium:proton	P	Inorganic ion transport and metabolism	Metabolism
677769_788982_gpsA_glycerol-3-phosphate	I	Lipid transport and metabolism	Metabolism
688237_799900_cysS_cysteinyl-tRNA	J	Translation, ribosomal structure and biogenesis	Information storage and processing
709030_821705_CFVT_0811_alpha/beta	S	Function unknown	Poorly characterized
713204_825879_pta_phosphate	C	Energy production and conversion	Metabolism
714699_827374_CFVT_0816_DUF305	S	Function unknown	Poorly characterized
72154_75015_fliD_flagellar	N	Cell motility	Cellular processes and signaling
722598_835288_CFVT_0825_endonuclease/exonuclease/phosphatase	S	Function unknown	Poorly characterized
745673_865858_CFVT_0861_RseP-like	M	Cell wall/membrane/envelope biogenesis	Cellular processes and signaling
750272_870457_750581_870766_750584_870769_750601_870786_750656_870841_CFVT_0866_putative	NU		Cellular processes and signaling
809995_931788_CFVT_0925_AsmA	M	Cell wall/membrane/envelope biogenesis	Cellular processes and signaling
814691_936484_hypB_hydrogenase	KO		Multiple
855307_979664_CFVT_0971_short-chain	S	Function unknown	Poorly characterized
864687_1001005_864721_1001089_CFVT_0986_DUF3519	-		Unclassified
865378_1001746_ctb_group	S	Function unknown	Poorly characterized
868219_1004597_acnB_aconitate	C	Energy production and conversion	Metabolism
871555_1007933_ribB_3,4-dihydroxy-2-butanone-4-phosphate	H	Coenzyme transport and metabolism	Metabolism

Query	COG category	COG functional classification	COG general categories
874971_1011349_era_GTP-binding	S	Function unknown	Poorly characterized
877290_1014045_CFVT_1000_hypothetical	-		Unclassified
878437_1015192_ctsD_transformation	NU		Cellular processes and signaling
885295_1022211_CFVT_1007_MCP-domain	NT		Cellular processes and signaling
886089_1023005_CFVT_1009_exopolyphosphatase,	FP		Metabolism
890677_1027593_CFVT_1016_transporter,	E	Amino acid transport and metabolism	Metabolism
892158_1029101_ansB_asparaginase	EJ		Multiple
919675_1057147_CFVT_1047_hypothetical	-		Unclassified
924848_1062324_lon_DNA-binding,	O	Post-translational modification, protein turnover, and chaperones	Cellular processes and signaling
932160_1069733_fucP_L-fucose	G	Carbohydrate transport and metabolism	Metabolism
938813_1077057_cmoB_tRNA	J	Translation, ribosomal structure and biogenesis	Information storage and processing
946534_1084785_CFVT_1074_putative	S	Function unknown	Poorly characterized
949387_1087650_dsbD_thiol:disulfide	CO		Multiple
953934_1092225_grpE_DnaK	O	Post-translational modification, protein turnover, and chaperones	Cellular processes and signaling
9551_9539_flgE_flagellar	N	Cell motility	Cellular processes and signaling
964164_1103004_potC_iron(III)/spermidine/putrescine	P	Inorganic ion transport and metabolism	Metabolism
969998_1108877_ligA_DNA	L	Replication, recombination and repair	Information storage and processing
991570_1130765_racS_two-component	T	Signal transduction mechanisms	Cellular processes and signaling

Table S10. Presence of the 7 peptidoglycan SNPs in 58 RefSeq annotated *Campylobacter fetus* strains, which reliably divided the assemblies into 2 groups.

**Assemblies**	**mraY (minus sense)**	**cysS (minus sense)**	**flgG (plus sense)**	**mfd (plus sense)**	**MutsS (plus sense)**	**rpoB (minus sense)**	**rpoC (minus sense)**
Campylobacter fetus* subsp. *fetus							
GCF_003426005.1	G	G	A	C	C	G	C
GCF_017896405.1	G	G	A	C	C	G	C
GCF_900475935.1	G	G	A	C	C	G	C
GCF_000015085.1	G	G	A	C	C	G	C
GCF_020828935.1	G	G	A	C	C	G	C
GCF_008014295.1	G	G	A	C	C	G	C
GCF_001699505.1	G	G	A	C	C	G	C
GCF_007723545.1	G	G	A	C	C	G	C
GCF_003426015.1	G	G	A	C	C	G	C
GCF_000759485.1	G	G	A	C	C	G	C
GCF_001399955.1	G	G	A	C	C	G	C
GCF_013406925.1	A	A	G	T	T	A	T
GCF_008527615.1	A	A	G	T	T	A	T
GCF_008526335.1	A	A	G	T	T	A	T
GCF_017896385.1	G	G	A	C	C	G	C
GCF_008693125.1	G	G	A	C	C	G	C
GCF_001699575.1	G	G	A	C	C	G	C
CP100646	G	G	A	C	C	G	C
CP100645	G	G	A	C	C	G	C
GCA_030544625.1	G	G	A	C	C	G	C
GCA_030544645.1	G	G	A	C	C	G	C
GCA_011600855.2	G	G	A	C	C	G	C
GCA_011600945.2	G	G	A	C	C	G	C
GCA_011600995.2	G	G	A	C	C	G	C
Campylobacter fetus* subsp. *venerealis							
GCF_001699685.1	A	A	G	T	T	A	T
GCF_016406645.1	G	G	A	C	C	G	C
GCF_016612875.1	A	A	G	T	T	A	T
GCF_016612955.1	A	A	G	T	T	A	T
GCF_016612945.1	A	A	G	T	T	A	T
GCF_000744035.1	A	A	G	T	T	A	T
GCF_000744025.1	A	A	G	T	T	A	T
GCF_000759515.1	A	A	G	T	T	A	T
GCF_016612925.1	A	A	G	T	T	A	T
GCF_030544605.1	A	A	G	T	T	A	T
GCF_001699745.1	A	A	G	T	T	A	T
GCF_008271385.1	A	A	G	T	T	A	T

Assemblies	mraY (minus sense)	cysS (minus sense)	flgG (plus sense)	mfd (plus sense)	MutsS (plus sense)	rpoB (minus sense)	rpoC (minus sense)
GCF_001699735.1	A	A	G	T	T	A	T
GCF_002592365.1	A	A	G	T	T	A	T
GCF_008526355.1	A	A	G	T	T	A	T
GCF_001699565.1	A	A	G	T	T	A	T
GCF_002592335.1	A	A	G	T	T	A	T
GCF_013406955.1	A	A	G	T	T	A	T
GCF_012274465.1	A	A	G	T	T	A	T
GCF_001686885.1	A	A	G	T	T	A	T
GCF_016612985.1	A	A	G	T	T	A	T
GCF_030544565.1	A	A	G	T	T	A	T
GCF_030544545.1	A	A	G	T	T	A	T
GCF_000512745.2	A	A	G	T	T	A	T
GCF_030544585.1	A	A	G	T	T	A	T
GCF_001699615.1	A	A	G	T	T	A	T
GCF_000967135.1	A	A	G	T	T	A	T
GCF_001699645.1	A	A	G	T	T	A	T
GCF_000414135.1	A	A	G	T	T	A	T
CP075536-CP075537	A	A	G	T	T	A	T
GCA_011600845.2	A	A	G	T	T	A	T
GCA_011600955.2	A	A	G	T	T	A	T
GCA_011601005.2	A	A	G	T	T	A	T
GCA_011601375.2	A	A	G	T	T	A	T

Figure S1: KEGG pathway "Peptidoglycan biosynthesis" (map00550), red box indicates *murC*, green box indicates *mraY* and blue boxes indicate *ftsI*.

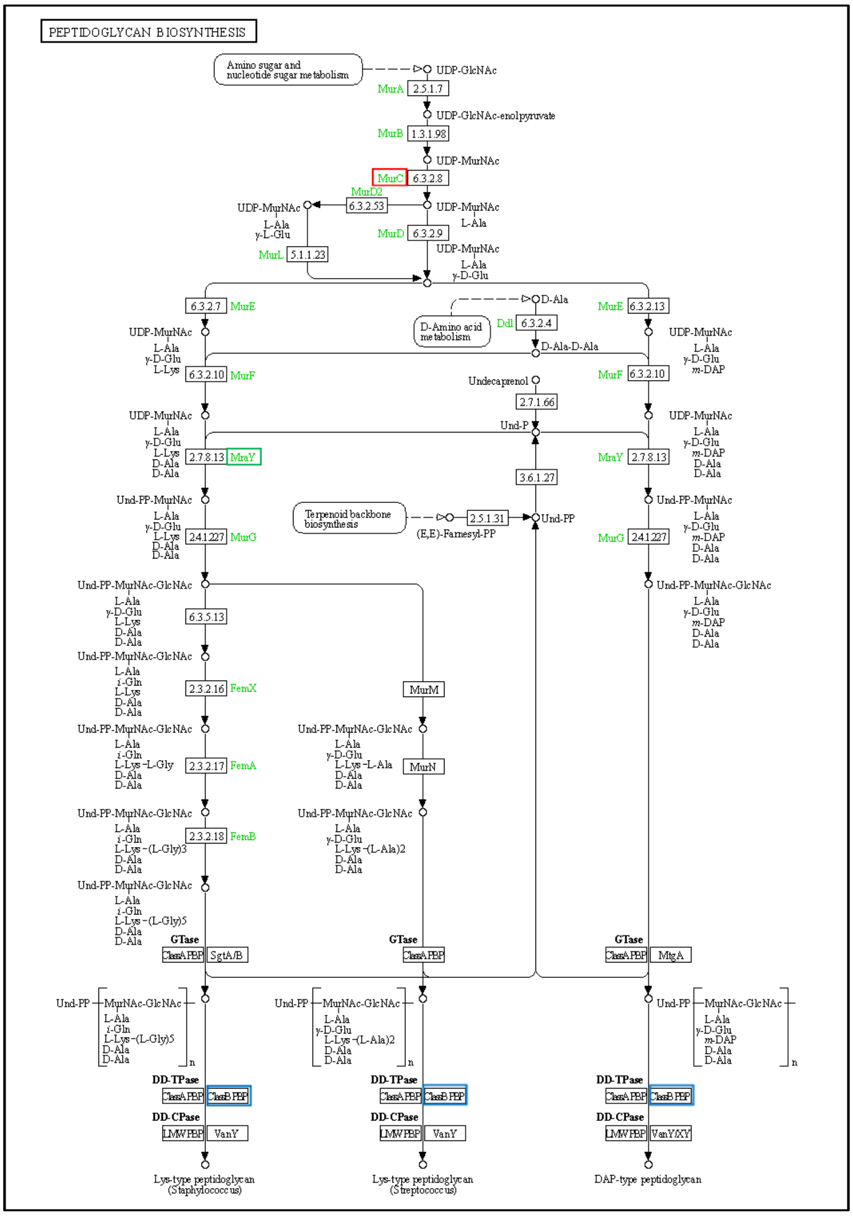

Table S8: List biovar single nucleotide polymorphisms (SNPs) which were differentiative between *Campylobacter fetus venerealis* and *Campylobacter fetus venerealis* bv. intermedius in Australian and non-Australian strains.

				**Australian strains**		**Non-Australian strains**		
**SNP position**	**Gene/CDS**	**Product**	**Type**	*C. fetus venerealis*	*C. fetus venerealis* bv. intermedius	*C. fetus venerealis*	*C. fetus venerealis* bv. intermedius	*C. fetus fetus*
232799	lysA	diaminopimelate decarboxylase	Missense	G	G	T	G	G
292839	yedF	selenium metabolism protein	Synonymous	G	G	T	G	G
380065	noncoding		Missense	A	A	C	A	A
429904	sapA7	surface array protein A	frameshift	C	C	T	N	T
784274	CFVT_0898	phosphatase, PhoX family (DUF839 domain)	Upstream gene variant	G	G	A	G	G
822843	CFVT_0937	[Ni-Fe] hydrogenase, small subunit HydA	Stop gain	G	G	A	G	G
913310	CFVT_1040	DUF3972 domain-containing protein	Missense	G	G	A	G	G
1425819	CFVT_1688	hypothetical protein	Synonymous	T	C	T	T	T
